# MITO-Luc/GFP zebrafish model to assess spatial and temporal evolution of cell proliferation in vivo

**DOI:** 10.1038/s41598-020-79530-5

**Published:** 2021-01-12

**Authors:** Luisa de Latouliere, Isabella Manni, Laura Ferrari, Federica Pisati, Maria Grazia Totaro, Aymone Gurtner, Emanuele Marra, Lucrezia Pacello, Ombretta Pozzoli, Luigi Aurisicchio, Maurizio C. Capogrossi, Gianluca Deflorian, Giulia Piaggio

**Affiliations:** 1 UOSD SAFU, IRCCS - Regina Elena National Cancer Institute, Via Elio Chianesi 53, 00144 Rome, Italy; 2 IFOM - FIRC Institute of Molecular Oncology, Milan, Italy; 3 Histopathology Unit, Cogentech S.C.a.R.L, 20139 Milan, Italy; 4 Institute of Translational Pharmacology, National Research Council, Rome, Italy; 5 Takis s.r.l., via Castel Romano 100, 00128 Rome, Italy; 6 VITARES -APS, via Castel Romano 100, 00128 Rome, Italy; 7 Laboratorio Di Biologia Vascolare e Medicina Rigenerativa - Centro Cardiologico Monzino - IRCCS (Istituto Di Ricovero E Cura a Carattere Scientifico), Milan, Italy; 8 Johns Hopkins University School of Medicine, Division of Cardiology, 301 Building, Suite 2400, 4940 Eastern Avenue, Baltimore, MD 21224 USA; 9 Laboratory of Cardiovascular Sciences, National Institute on Aging/National Institutes of Health, Baltimore, MD 21224 USA; 10 Cogentech SRL - Benefit Corporation, Milan, Italy; 11 Present Address: Pfizer Italia, Via A.M. Mozzoni 12, 20152 Milan, Italy

**Keywords:** Regeneration, Genetic models, Bioluminescence imaging, Confocal microscopy, Cell growth

## Abstract

We developed a novel reporter transgenic zebrafish model called MITO-Luc/GFP zebrafish in which GFP and luciferase expression are under the control of the master regulator of proliferation NF-Y. In MITO-Luc/GFP zebrafish it is possible to visualize cell proliferation in vivo by fluorescence and bioluminescence. In this animal model, GFP and luciferase expression occur in early living embryos, becoming tissue specific in juvenile and adult zebrafish. By in vitro and ex vivo experiments we demonstrate that luciferase activity in adult animals occurs in intestine, kidney and gonads, where detectable proliferating cells are located. Further, by time lapse experiments in live embryos, we observed a wave of GFP positive cells following fin clip. In adult zebrafish, in addition to a bright bioluminescence signal on the regenerating tail, an early unexpected signal coming from the kidney occurs indicating not only a fin cell proliferation, but also a systemic response to tissue damage. Finally, we observed that luciferase activity was inhibited by anti-proliferative interventions, i.e. 5FU, cell cycle inhibitors and X-Rays. In conclusion, MITO-Luc/GFP zebrafish is a novel animal model that may be crucial to assess the spatial and temporal evolution of cell proliferation in vivo.

## Introduction

*Danio rerio* (zebrafish) represents a versatile animal model to study a variety of biologic processes including cancer growth and its response to treatment^1,2^, cardiac damage and regeneration^3,4^, wound healing, and angiogenesis^5,6^. Indeed, it represents a powerful experimental tool, intermediate between cell culture and mammalian animal models and, ultimately, human clinical trials. Compared to other in vivo models, zebrafish is highly attractive due to the small size, embryo optical transparency, low maintenance cost, high fecundity and rapid development. The complete genomic sequence and various tools for genetic manipulation are available^7–10^. Furthermore, cell and molecular processes such as proliferation, differentiation, apoptosis and signaling pathways are highly conserved and about 70% of all human disease genes have functional homologs in zebrafish^11^. On this basis, several mutant and transgenic lines have been generated, in order to shed light on the genes and/or molecular mechanisms involved in human diseases^12–14^. Although fluorescent reporters are valuable optical tools for real-time imaging in transparent zebrafish embryos^15^ they are not useful in living adult animals because zebrafish adult skin is not transparent anymore and therefore tissue autofluorescence makes the signal-to-noise ratio very low; a transparent adult zebrafish model is now available. This represents an extremely useful tool for cell transplantation analysis in the adult zebrafish^16^.

Non-invasive BioLuminescence Imaging (BLI) in living small animals represents an interesting and powerful approach to molecular imaging^17^. The luciferase-luciferine reaction can be linked to a specific biological process allowing its real-time non-invasive imaging. Non-invasive BLI has been widely used in mice to monitor gene expression^18^, bacterial and viral infections^19^, as well as cell proliferation and transformation^20^. This technique allows the serial quantification of spatial and temporal evolution of the process of interest in the same animal^19^. This reduces the number of required animals and enhances the scientific validity of the results because each animal serves as its own control. Luciferase light detection in an intact animal is dependent upon many parameters including the number and depth of the luciferase-labeled cells within the body and light attenuation by all tissue between light-emitting cells and the detection system. In zebrafish, these issues do have a minor impact due to the model’s small size. To date, few examples of zebrafish luciferase reporter models, with ubiquitously or tissue-specific luciferase expression, have been described^21^.

We previously generated a mouse model, called MITO-Luc reporter mouse, to visualize proliferation events in live animals. In these lines the luciferase reporter gene is under the control of a cyclin B2 minimal promoter containing three CCAAT boxes conserved between mouse and human and tightly regulated by the nuclear transcription factor Y (NF-Y)^22^. The trimeric CCAAT-binding NF-Y transcription factor consists of three subunits, NF-YA, NF-YB, and NF-YC all required for DNA binding^23^. NF-Y is conserved in evolution and its activity is required for transcription of regulatory genes responsible for cell cycle progression, thus supporting cell proliferation^24–33^.

Here we describe the generation and characterization of a new zebrafish model, called MITO-Luc/GFP, in which green fluorescent protein (GFP) and luciferase reporter genes are placed under transcriptional control of the same proliferation-dependent promoter fragment, as in mouse model. This versatile tool consents to choose the most appropriated method of analysis. Due to the optical transparency, cell proliferation can be analyzed in live embryos by GFP, while in live adult animals luciferase activity allows the quantitative assessment and visualization of cell proliferation in deep tissues by BLI. This new model is a remarkable tool for monitoring cell proliferation in longitudinal studies along the entire zebrafish life.

## Results

### Establishing *Tg (cyclin B2:Luc/GFP)* MITO-Luc/GFP transgenic zebrafish lines

**Figure 1:**
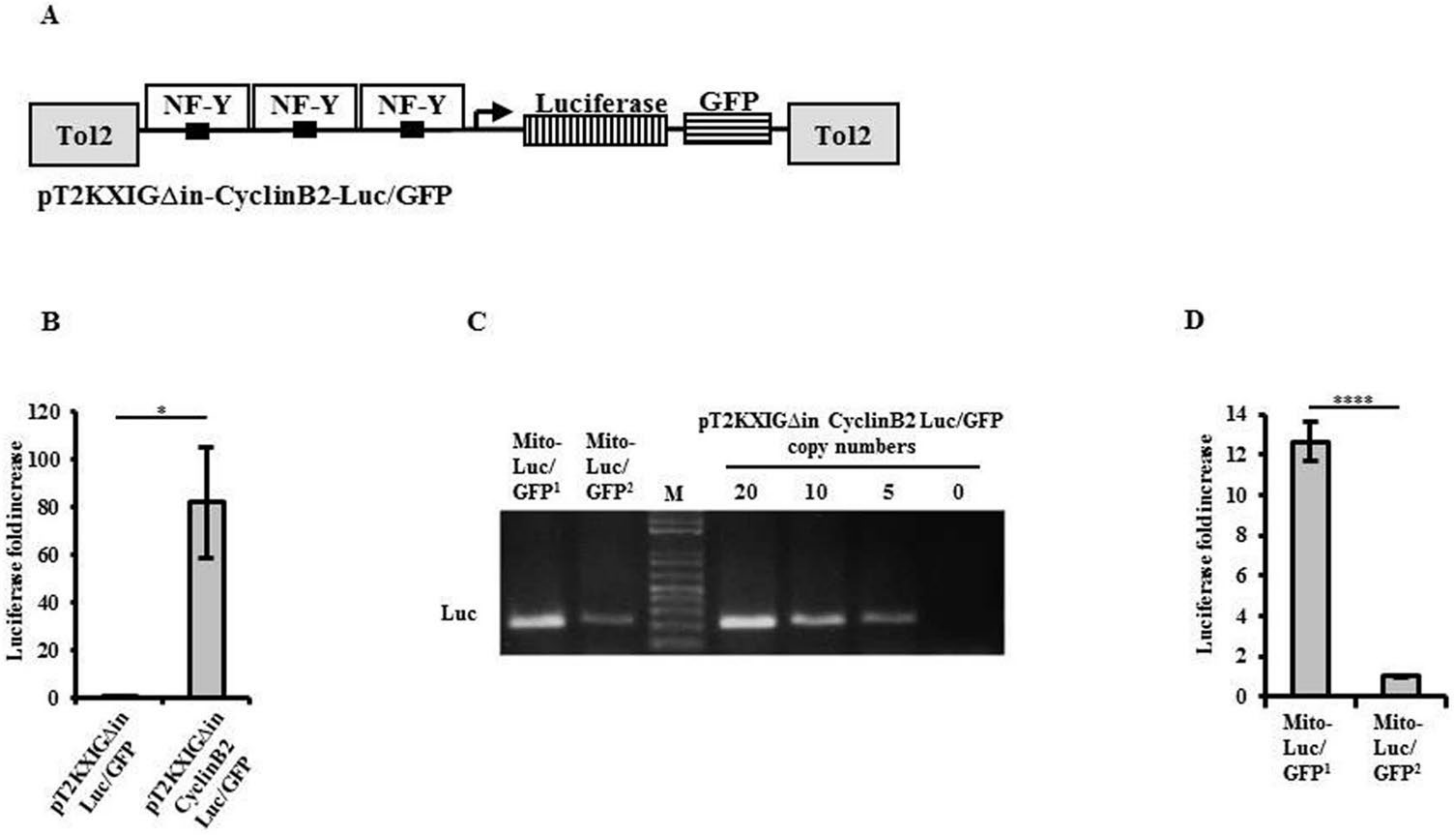
Establishing Tg (cyclin B2:Luc/GFP) MITO-Luc/GFP transgenic zebrafish lines. (**A**) Schematic representation of the pT2KXIGΔin-cyclin B2-Luc/GFP expression vector. A fragment of murine cyclin B2 promoter containing CCAAT boxes (black) was cloned upstream of the firefly luciferase gene (vertical stripes) and the green fluorescent protein GFP (horizontal stripes), in the pT2KXIGΔin plasmid containing the Tol 2 transposable element (gray). The binding of NF-Y to the CCAT boxes is shown. (**B**) Relative luciferase activity obtained in one to four-cell stage AB embryos transiently injected with the pT2KXIGΔin-Luc/GFP or the pT2KXIGΔin-cyclin B2-Luc/GFP. The error bars are mean standard deviations from two experiments performed in duplicate. Asterisks denote significant differences between pT2KXIGΔin-cyclin B2-Luc/GFP and empty vector assessed by t-test (*p < 0.05). (**C**) Quantification of transgene copy number in MITO-Luc/GFP zebrafish lines by quantitative PCR. Amplification of 10/15 transgene copies from MITO-Luc/GFP^1^ and 1 from MITO-Luc/GFP^2^ genomic DNA, is quantified by comparing amplifications from known pT2KXIGΔin-cyclin B2-Luc/GFP copy numbers. Full-length gel is presented in Supplementary Figure S17. (**D**) Relative luciferase activity in MITO-Luc/GFP^1^ and MITO-Luc/GFP^2^ zebrafish lines. The error bars are mean standard deviations from two experiments performed at least in triplicate with 3 embryos in each group. Asterisks denote significant differences between MITO-Luc/GFP^1^ and MITO-Luc/GFP^2^ embryos assessed by t-test (****p < 0.0001).

To develop a zebrafish model to visualize proliferation events in the whole animal, the promoter fragment used in MITO-Luc reporter mice^22^ was cloned upstream the sequences of firefly luciferase (Luc) and GFP reporter genes within the zebrafish Tol2 transposable element pT2KXIG plasmid (Fig. 1A). We used the mouse promoter since the three NF-Y subunits are highly conserved between mouse and zebrafish^34^(Supplementary Table S1). Moreover, zebrafish cyclin B2 promoter contains two of the three CCAAT boxes present on the fragment of the murine promoter^22^(Supplementary Fig. S1), thus indicating that murine cyclin B2 promoter region may be sensitive to NF-Y activity also in zebrafish. First, we tested weather the resulting pT2KXIGΔin-cyclin B2-Luc/GFP plasmid was actually a read out of proliferation. To this aim we transiently transfected with pT2KXIGΔin-cyclin B2-Luc/GFP plasmid, a mouse myoblast cell line that allows to easily observed mitotic and post-mitotic states^35^ as confirmed by a immunofluorescence microscopy confocal analysis after 3 h BrdU pulse (Supplementary Figure S2). In vitro luciferase activity assay showed 14.5-fold increased transcriptional activity in mitotic cells compared to post-mitotic ones (Supplementary Figure S3) thus proving the specificity of this reporter system for proliferating cells. To investigate the activity of pT2KXIGΔin-cyclin B2-Luc/GFP construct in zebrafish it was transiently injected into one to four-cell stage wild type embryos and, as control, the pT2KXIGΔin-Luc/GFP empty construct was transfected, too. In vitro luciferase activity assay performed in lysates showed 80-fold increased transcriptional activity in pT2KXIGΔin-cyclin B2-Luc/GFP compared to pT2KXIGΔin-Luc/GFP injected embryos (Fig. 1B). These experiments demonstrate a murine cyclin B2 promoter-dependent luciferase expression in injected zebrafish embryos.

To generate stable lines, the pT2KXIGΔin-cyclin B2-Luc/GFP construct and Transposase mRNA were co-injected into one to four-cell stage wild type embryos. Transgenic founders were collected by using GFP fluorescence microscopy analysis. Two independent transgenic zebrafish lines were identified and named MITO-Luc/GFP^1^ and MITO-Luc/GFP^2^. Quantitative PCR analysis indicated a different number of the transgene copies in the two lines (Fig. 1C). Consistent with these results, MITO-Luc/GFP^1^ expresses more in vitro luciferase activity than MITO-Luc/GFP^2^ embryos (Fig. 1D) and in vivo confocal microscopy shows a higher fluorescence level in MITO-Luc/GFP^1^ vs MITO-Luc/GFP^2^ embryos (Supplementary Fig. S4); finally, Southern Blotting assay confirmed multiple transgene insertions in MITO-Luc/GFP^1^ (Supplementary Fig. S5). In contrast, inverse PCR^36^ in MITO-Luc/GFP^2^ (Supplementary Fig. S6) indicated a single transgene insertion located in the first intron of the AMP-activated, gamma 2 non-catalytic subunit protein kinase gene (*prkag2a*) on chromosome 24, position (28.173.305–28.302.316)(ZFIN Gbrowse). These results are consistent with the quantitative PCR data (Fig. 1C). PCR on cDNAs obtained from transgenic lines show that both luciferase and GFP transcripts are present on a single transcript (Supplementary Fig. S7). On the contrary western blot on protein extracts from transgenic embryos show that the proteins are two (Supplementary Fig. S8). Sequence of the construct shows that the two open reading frames are separated by two CC (Supplementary Figure S9) suggesting that a leaky scanning translation occurs on the transcript, leading to luciferase and GFP proteins expression from the same mRNA^37^.

### Fluorescence and bioluminescence correlate with proliferation in MITO-Luc/GFP zebrafish strain

**Figure 2:**
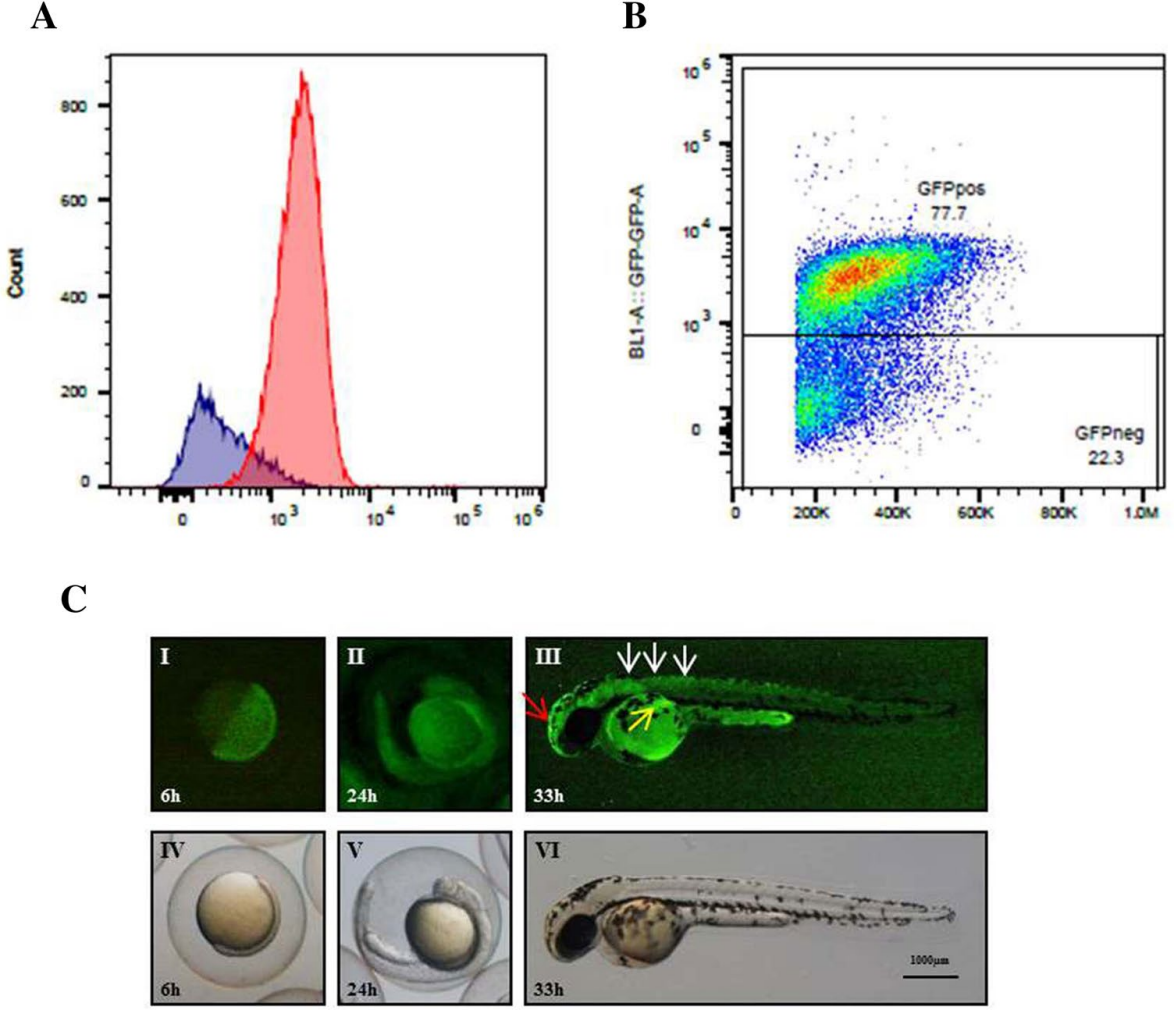
Fluorescence correlates with proliferation in MITO-Luc/GFP zebrafish line. (**A**) Flow cytometry analysis of cells dissociated from 200 zebrafish MITO-Luc/GFP 40 hpf embryos, previously incubated for 3 h with BrdU. Fixed cells were stained with anti-BrdU and anti-GFP antibodies. Histogram plot represents different BrdU accumulation in GFP positive (red) and negative (blue) cells. (**B**) GFP positive and GFP negative cells gating strategy. (**C**) GFP signal in 6 (I), 24 (II) and 33 hpf (III) embryos. GFP expression is almost ubiquitous throughout 6 and 24 hpf embryos (I,II). It is tissue specific in 33 hpf embryos being accumulated in the head (III, red arrow), proximal trunk (III, white arrows) and in developing fin (III, yellow arrow). Bright light images are shown (IV,V,VI).

To evaluate the reliability of MITO-Luc/GFP zebrafish lines as a new tool to visualize cell proliferation through GFP fluorescent signal, we focused on the MITO-Luc/GFP^1^ which expresses the higher number of inserted transgenes. FACS analysis performed on cells derived from about 200 transgenic embryos at 40 hpf, subjected to a 3 h BrdU pulse, showed a high predominance of BrdU accumulation (Fig. 2A) in GFP positive gated population rather than in GFP negative gated population (Fig. 2B), indicating that in this transgenic strain GFP is mostly expressed in proliferating cells. As expected, not all GFP positive cells have incorporated BrdU. This is due to the half-life of the GFP protein which, being 26 h^38^, also traces cells that have stopped to proliferate in the previous 26 h.

Next, GFP expression was observed at 6, 24 and 33 h post fertilization (hpf) embryos by fluorescent microscopy (Fig. 2C). At early stages (Fig. 2C I,II) the GFP expression is almost ubiquitous throughout the embryo; in contrast, it appears to be tissue specific in 33 hpf embryo where it is detected in the head (Fig. 2C III, red arrow), proximal trunk (Fig. 2C III, white arrows) and in developing fin (Fig. 2C III, yellow arrow). A background signal coming from the yolk sac is due to auto-fluorescence as demonstrated in wild-type fishes (Supplementary Fig. S10). Interestingly, this picture recapitulates published data on the recruitment of BrdU in 36 hpf embryos exposed to pulses of this DNA analog^39^.

Next, using dechorionated and low-melting agarose embedded live 4,5 hpf embryos, sequential stages of embryonic development were captured by confocal microscopy (until 50% epiboly stage)^40^. GFP-positive cells were identified along the entire acquisition (Supplementary Fig. S11) and different cell layers were distinguished (Supplementary movie S1 online). Morphogenetic cell movements of extension that characterize the early development of zebrafish embryos from 30 to 50% epiboly were also detected (Supplementary Fig. S11). Of note, from fertilization to 50% epiboly stage, cells divide continuously thus indicating that GFP positive are proliferating cells.

Similarly, 19 hpf embryos were imaged until the 26 somites stage. In Supplementary movie S2 online, somites development and eye enlargement are visible and origin of the primary organs and constriction of yolk extension are apparent. At this stage several cells are dividing rapidly: the brain and lens develop, neurons and muscle precursors establish and the tail elongates^40^, confirming that GFP positive are proliferating cells.

**Figure 3:**
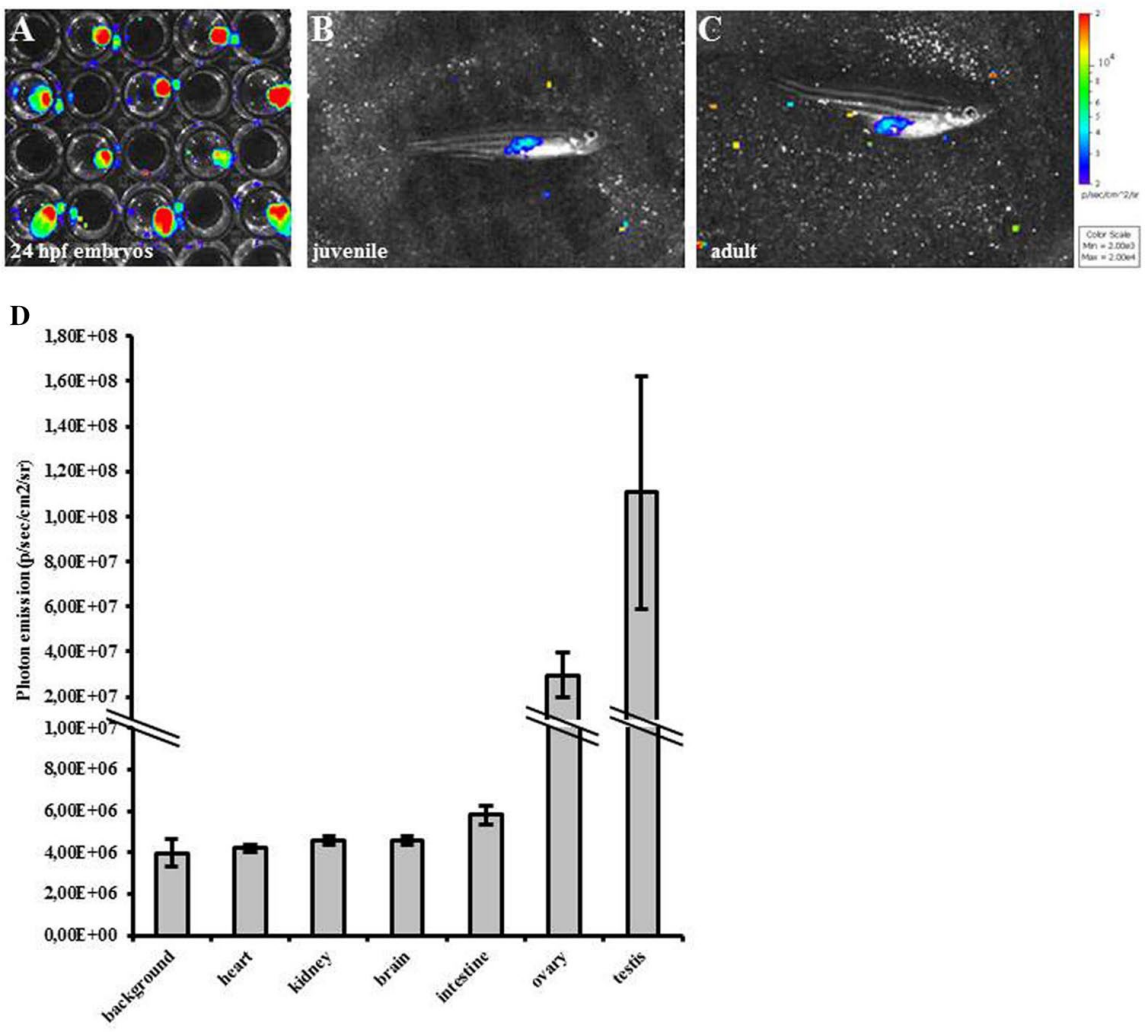
Bioluminescence correlates with proliferation in MITO-Luc/GFP zebrafish line. (**A–C**) In vivo BLI of representative MITO-Luc/GFP^1^ zebrafish embryos (1 embryo for each well) (**A**), juvenile (**B**) and adult (**C**) stages. Light emitted from the animal appears in pseudocolor scaling. (**D**) Photon emission quantification of organs extracted from adult zebrafish. The error bars are mean standard deviations from six samples for each organ.

Additional experiments were performed to establish whether physiologic proliferation events could be visualized in whole animals. Embryos, juveniles and adults were collected, anesthetized, bathed in D-luciferin and in vivo imaged (Fig. 3A–C). One embryo per well was imaged at 24 hpf stage. BLI demonstrated a signal from the entire embryo, as expected at this stage of development, when all cells are highly proliferating (Fig. 3A). Of note, this result confirms GFP expression analysis at the same stage. In juvenile and adult animals light is much more specifically localized in the abdomen (Fig. 3B,C). Luciferase enzymatic activity was also measured in homogenates from embryo, juvenile and adult MITO-Luc/GFP^1^ zebrafish; AB wild-type strain was used as negative control (Supplementary Fig. S12). The in vitro results closely resemble those seen in vivo (Supplementary Fig. S13), being luciferase activity higher in embryo than in juvenile and adult homogenates. Unexpectedly, luciferase activity was more pronounced in juvenile than in adult fish. This result can be explained considering proliferation/total tissue ratio. In fact, being juveniles smaller than adults, in in vitro experiments, proliferating cells are less diluted in the homogenates from the entire animal. This does not happen in vivo, where only the signal coming from proliferating tissue is detected.

To elucidate which abdominal organs are the source of light seen in vivo by BLI, photon emission was measured ex vivo*,* in explanted organs from both male and female adult fishes (Fig. 3D). High luciferase activity was detected in gonads, where a high number of proliferating cells are present^41,42^. Although to a lesser extent, also other organs produce light: brain, kidney and intestine (these organs are known to include a low number of proliferating cells)^43–46^. As expected, heart is negative and comparable to background, since, in adult animals, under baseline conditions, myocardial cell proliferation does not occur.

### Markers of proliferating cells co-localize with luciferase and GFP in MITO-Luc/GFP zebrafish tissues

**Figure 4:**
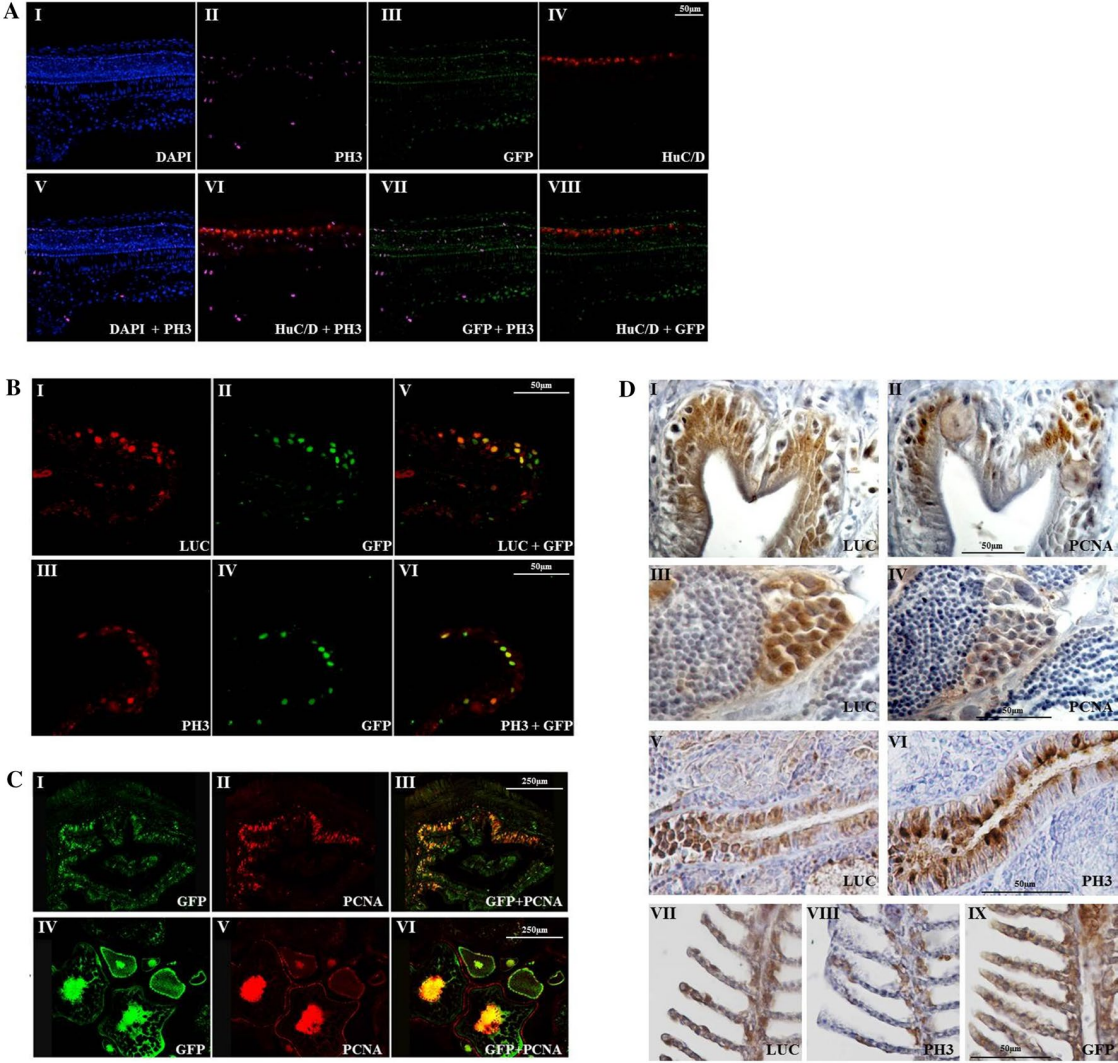
Proliferation markers colocalize with GFP and luciferase proteins in MITO-Luc/GFP zebrafish tissues*.* (**A**) Immunofluorescence analysis of DAPI (I), pH3 (II), GFP (III), HuC/D(IV), DAPI-pH3 merge (V), pH3-HuC/D merge (VI), pH3-GFP merge (VII) and GFP-HuC/D merge (VIII) in the trunk region of 24 hpf whole-mount transgenic embryos. (**B**) Immunofluorescence analysis of luciferase (I), GFP (II, IV), pH3 (III), luciferase-GFP merge (V) and pH3-GFP merge (IV) in adult epidermal tissue. (**C**) Immunofluorescence analysis of GFP (I,IV), PCNA (II,V), GFP-PCNA merge (III,VI), in intestine (I,II,III) and ovary (IV,V,VI). (**D**) Immunohistochemical analysis of luciferase (I, III, V, VII), PCNA (II, IV), pH3 (VI, VIII) and GFP (IX) in adult intestine (I, II), testes (III, IV), kidney (V, VI) and gills (VII, VIII, IX). In all tissue analyzed, cells expressing proliferation markers, either PCNA or pH3, show immunoreactivity for luciferase and/or GFP.

To verify the suitability of MITO-Luc/GFP^1^ zebrafish model as tracer of proliferation events the cellular distribution of luciferase and GFP were analysed by immunofluorescence. As expected, both reporters are highly expressed in almost all nuclei in the distal trunk section of 24 hpf embryos, being in this stage all cells highly proliferating (Supplementary Fig. S14). Likewise, in the trunk region of 24 hpf whole-mount transgenic embryos (Fig. 4A), several GFP positive cells are co-expressing the proliferation marker phosphorylated histone 3 isoform (pH3) a well-known marker of mitosis^47^(Fig. 4A II,III,VII).On the contrary, lack of overlapping signals between GFP and the post-mitotic neurons marker HuC/D is clearly observable (Fig. 4A III, IV, VIII). Overall, these data indicate that, in MITO-Luc/GFP zebrafish embryos, GFP is mostly expressed in proliferating cells while post-mitotic cells, like those expressing HuC/D marker, are all GFP negative.

Furthermore a colocalization of luciferase and GFP was observed also in adult epidermal fin tissue (Fig. 4B I,II,V) thus demonstrating that the two reporters are expressed in the same cells. Interestingly, several GFP positive cells also express pH3^47^(Fig. 4B III,IV,VI). In line with these results, the majority of GFP positive cells in intestine (Fig. 4C I,II,III) and in ovary (Fig. 4C IV,V,VI) also express proliferation cellular nuclear antigen (PCNA), another well-documented marker for cell proliferation in zebrafish^48^. Overall, these data indicate that GFP positive are proliferating cells while post-mitotic cells are GFP negative.

Although the majority of GFP positive cells are also positive for proliferation markers, not all of them are. One possible explanation is due to the longer half-life of GFP protein (26 hr)^38^ compared with that of luciferase^49^ and PCNA^50^. Regarding pH3 this is a specific mitotic marker^47^, thus limiting the percentage of colocalization with our tracer GFP expressed along all cell cycle phases.

Next, by Immunohistochemistry (IHC), sequential paraffin section from adult MITO-Luc/GFP^1^ tissues were stained with antibodies against luciferase, PCNA or pH3. Of note, in all analyzed tissues, cells are found that express proliferation markers, either PCNA or pH3, and also immunoreactivity for luciferase. Luciferase and PCNA are coexpressed in the majority of the cells of the intestinal villi (Fig. 4D I-II) and testis spermatogonia (Fig. 4D III-IV). Luciferase and pH3 are coexpressed in several cells of the nephron proximal tubule in kidney (Fig. 4D V-VI) as well as in gill filaments and lamellae (Fig. 4D VII-VIII). As expected, in this last case co-expression is not 100% as pH3 is a mitotic phase marker of the proliferating cells, as discussed above (Fig. 4D IX).

Taken together, these results demonstrate that both luciferase and GFP reporter genes are expressed in proliferating cells in MITO-Luc/GFP^1^ zebrafish, and suggest that this animal model is a powerful tool to visualize cell proliferation in live animals.

### Caudal fin regeneration in MITO-Luc/GFP zebrafish line

**Figure 5:**
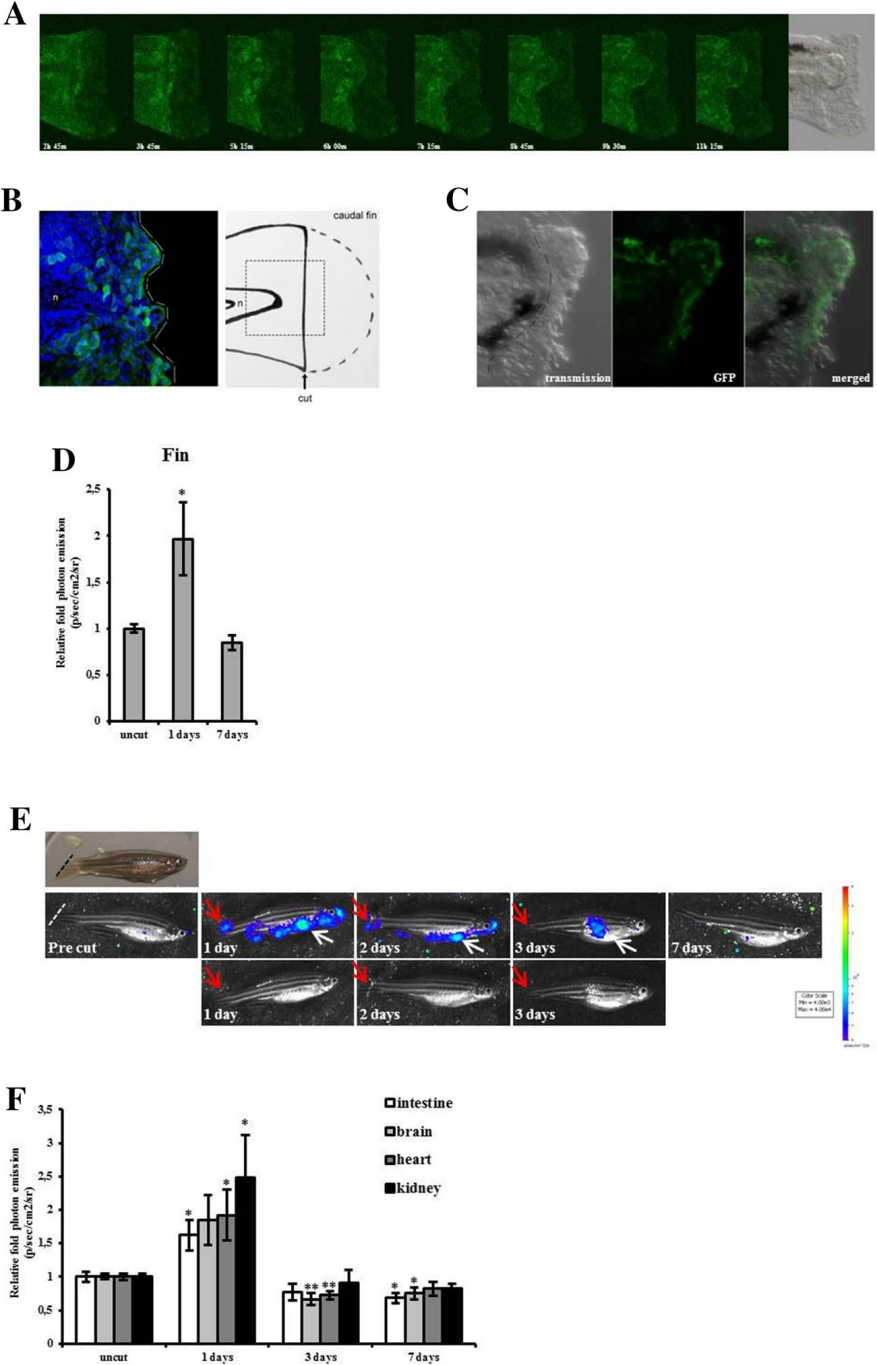
Caudal fin regeneration in MITO-Luc/GFP zebrafish. (**A**) Summary frame pictures of GFP during caudal fin regeneration in a 3 dpf embryo. The tridimensional images are the results of a merge of nine different layers acquisition (Z-stack). The dynamic of blastoma growth is clearly visible. Bright light image of the fin is shown in the last picture. (**B**) Immunofluorescence analysis of GFP-DAPI merge 1 day after fin clip in a 3 dpf embryo. The recruitment of GFP positive cells on the marginal edge is visible. Schematic representation of fin clip is shown. Notocord (n) is shown as anatomical reference. (**C**) Confocal imaging of GFP positive cells during regeneration of the caudal fin in a MITO-Luc/GFP^2^ 2 dpf live embryo. Dotted line in the left image indicates the amputation site, made 24 h before the acquisition. (**D**) Relative quantification of fin from adult zebrafish after fin clip. Luciferase activity was measured at 1 and 7 days after fin clip. Uncut fins were used as control. The error bars are mean standard deviations from six samples for each group. Asterisks denote significant differences between control and treated groups assessed by t-test (*p < 0.05). (**E**) BLI of representative MITO-Luc/GFP^1^ zebrafish longitudinal analysis (pre-cut, 1 day, 2 days, 3 days and 7 days) after fin clip procedure. Red arrows indicate light coming from regenerating caudal fin. White arrows indicate light coming from the abdominal organs. A bright picture and images without luminescence signal are shown for 1, 2 and 3 days as anatomical references. Light emitted from the animals appears in pseudocolor scaling. (**F**) Ex vivo BLI of zebrafish organs after fin clip. Relative fold induction of photon emission is presented. Luciferase activity was measured in dissected organs from zebrafish sacrificed 1, 3, and 7 days after fin clip. Uncut zebrafish were used as control group. The error bars are mean standard deviations from six fishes for each group. Asterisks denote significant differences between control and treated groups assessed by t-test (*p < 0.05, **p < 0.01).

These experiments were aimed at establishing whether cell proliferation could be visualized after inducing tissue damage in MITO-Luc/GFP zebrafish. For these experiments we took advantage of zebrafish ability to regenerate amputated fins, a process associated with cell proliferation^51^. Cell proliferation and migration contribute to the early regeneration of zebrafish fins; epithelial and mesenchymal cells proximal to the level of the amputation, few hours later the injury, are strongly labeled with BrdU^52^. The expression of GFP was imaged during regeneration of the caudal fin in 3 days post fertilization (dpf) MITO-Luc/GFP^1^ embryos after fin clip. Embryos were subjected to the cut of the distal segment of the caudal fin, embedded in agarose and imaged for 14 h (Supplementary movie S3 online). Imaging demonstrated growth of the blastoma (in Fig. 5A selected frame pictures from the movie). A rapid migration of GFP positive cells to the wound site is observed, thus indicating that these cells are proliferating to replace the wounded epidermis with new tissue. This result was confirmed by immunofluorescence analysis, where the recruitment of GFP positive cells on blastoma, 1 day after cut, is clearly detected (Fig. 5B). In agreement, 24 h after a caudal fin fragment dissection from MITO-Luc/GFP^2^ 2 dpf live embryos, many GFP positive cells are visible at the tip of the caudal fin, adjacent to the region of amputation (Fig. 5C). This experiment confirmed that, in both transgenic zebrafish line, GFP is expressed only in cells involved in proliferative events, such as caudal fin regeneration.

Next, we monitored the progress of caudal fin regeneration by BLI in adult animals after fin clip procedure. Three adult zebrafish males and three females were anesthetized and bathed in D-luciferin and ex vivo imaging of the caudal fin was performed; the animals were sacrificed 1 and 7 days after fin clip. Luciferase activity of the fin stump doubled 1 day after fin clip and was back to its baseline value one week after the procedure (Fig. 5D). We investigated this process also in vivo by BLI by serial imaging of the same animal following fin clip. After the amputation, fishes were placed on a water-soaked sponge and subjected to longitudinal in vivo imaging sessions; a representative experiment is shown in Fig. 5E. Images were obtained before fin clip (Fig. 5E, precut). The first day after the cut, the caudal fin stump emits intense light indicating that cell proliferation is occurring in that area (Fig. 5E 1 day, red arrow), whereas the emitted light decreases on the subsequent days. A week post amputation light emission detected in the regenerated fin has completely ceased (Fig. 5E 7 days). These observations are in agreement with published data on caudal fin regeneration showing that the wound healing process starts approximately 12 to 48 h post-amputation^51^ even if amputated fin grows for longer than a week to reach the original length, in the last part of the process few cells are proliferating and might not be seen, due to a matter of sensitivity of the model.

In parallel with the signal coming from caudal fin, we observed a systemic luciferase activation in the abdomen and head starting the first day after amputation (Fig. 5E 1 day, white arrow) and persisting at least for two additional days (Fig. 5E 2 days, 3 days). Light emission at all sites ceased and was completely undetectable one week after fin clip (Fig. 5E 7 days).

To further verify which organ/s emit/s light, ex vivo experiments were carried out to measure luciferase activity of dissected organs from three males and three females sacrificed 1, 3, and 7 days after fin clip. Luciferase activity was measured in dissected organs previously bathed in D-luciferin. Although to a different extent, high luciferase activity was detected in intestine, brain, heart and kidney dissected 1 day after fin clip (Fig. 5F). The gonads exhibited a slight increase in luciferase activity after fin clip as well, but these changes were not statistically significant (Supplementary Fig. S15).

Taken together, our data demonstrate that after fin clip, GFP and luciferase expression are induced in MITO-Luc/GFP^1^ zebrafish strain and strongly indicate that in this model system it is possible to dynamically visualize induction of proliferation events in specific organs of interest.

### Inhibition of luciferase activity in zebrafish embryos upon anti-proliferative treatments

**Figure 6:**
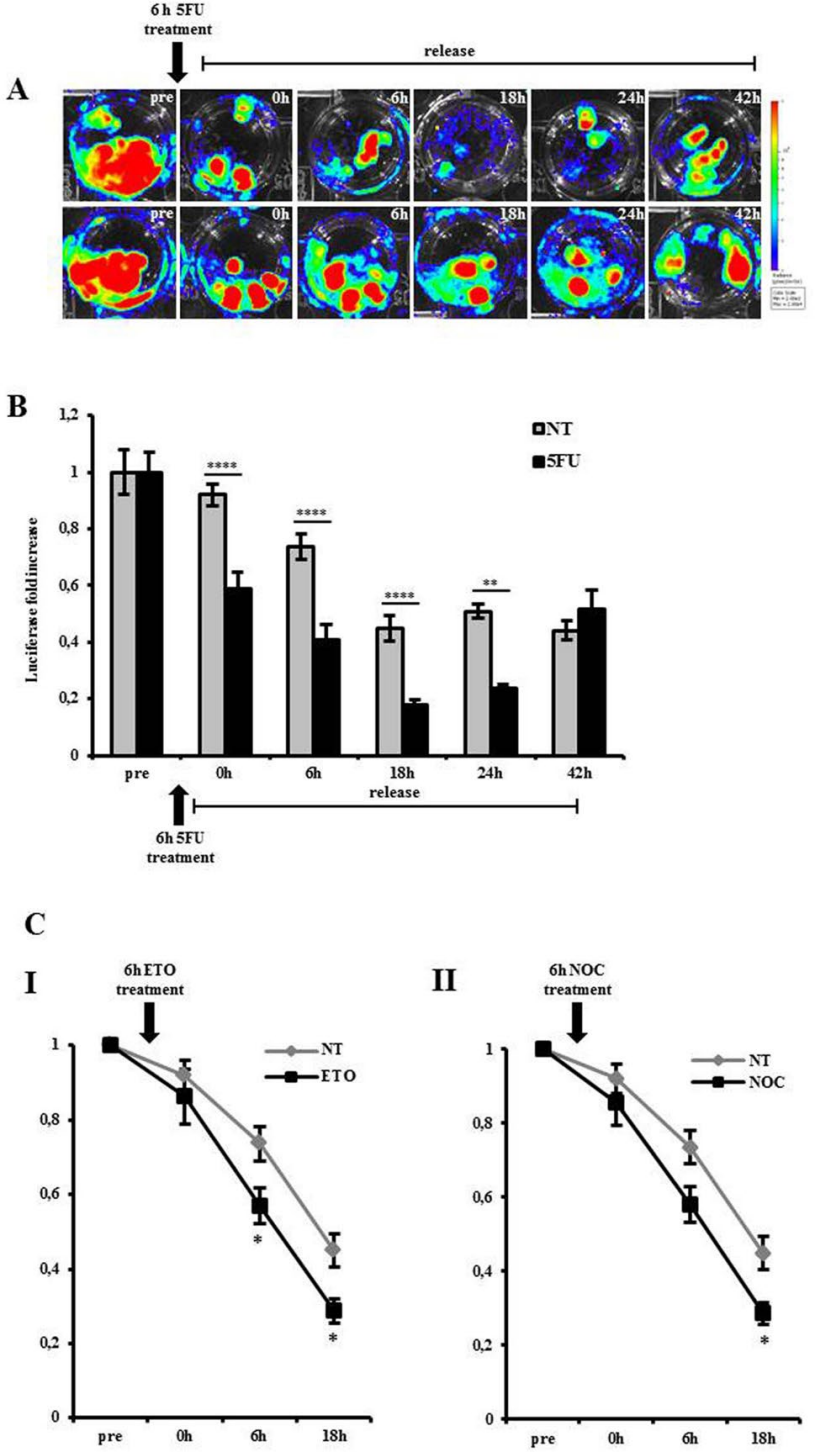
Inhibition of luciferase in zebrafish embryos upon antiproliferative treatments. (**A**) BLI of a pool of 10 MITO-Luc/GFP embryos before treatment (pre). The embryos have been treated with a pulse of 100 mM 5FU for 6 h as indicated. Upon 5FU washout and replacement with fresh water BLI was measured at time 0, 6, 18, 24 and 42 h. Bottom panel represent untreated control group imaged at the same time points. (**B**) Relative luciferase activity of a pool of 10 MITO-Luc/GFP embryo extracts collected before treatment. The embryos were treated with a pulse of 100 mM 5FU as indicated. Following 5FU washout and replacement with fresh water embryo extracts were collected at time 0, 6, 18, 24 and 42 h. Gray bars represent untreated control pool extracts collected at the same time points. The error bars are mean standard deviations from three experiments performed in triplicate. Asterisks denote significant differences between control and treated groups assessed by t-test (***p < 0.001****p < 0.0001). (**C**) Relative luciferase activity of a pool of 10 MITO-Luc/GFP embryo extracts. The embryos were treated with a pulse of 20 mM Etoposide (I) and 150 nM Nocodazole (II) for 6 h as indicated. Upon drug removal and replacement with fresh water embryo extracts were collected at time 0, 6, 18 h. Gray lines represent untreated control pool extracts collected at the same time points. The error bars are mean standard deviations from three experiments. Asterisks denote significant differences between control and treated groups assessed by t-test (*p < 0.05).

We investigated whether inhibition of cell proliferation was associated with inhibition of luciferase expression in MITO-Luc/GFP^1^ zebrafish model. To this aim, a pool of 24 hpf embryos was treated with a sub-lethal dose of a well-known anti-proliferative drug, 5Fluoro Uracil (5FU)^53^. We acquired images of the embryos just before treatment (Fig. 6A, pre). 5FU was dissolved in water containing dechorionated 24 hpf embryos and replaced with fresh water after 6hrs (Fig. 6A, 0 h). Treated embryos were then analyzed in vivo through imaging sessions at sequential timing. After 6, 18 and 24 h treatment we observed a strong inhibition of luciferase expression compared to untreated embryos (Fig. 6A, 6 h, 18 h, 24 h). The signal of 5FU-treated embryos progressively increased after removal of the drug and, after 42 h it was comparable to control (Fig. 6A, 42 h). Luciferase in vitro enzymatic assay, performed on embryos lysates, confirmed the in vivo results (Fig. 6B).

In additional experiments, 24 hpf embryos were irradiated using sub-lethal X-rays at different doses^54^. Luciferase activity of untreated, 1.8 Gy and 2.7 Gy treated embryos was measured in vitro*,* 6 h after irradiation. As shown in supplementary figure S16, X-ray radiation determined a reproducible decrease of luciferase activity in irradiated groups, compared with not-irradiated controls.

To further support the hypothesis that MITO-Luc/GFP zebrafish may be useful in large-scale drug screening experiments, the effect of Etoposide and Nocodazole, two cell cycle inhibitors, was investigated in 24 hpf embryos treated with sub lethal doses of these drugs^55,56^. We found that both cell cycle inhibitors markedly decreased luciferase activity compared to untreated control embryos (Fig. 6C I,II). As expected, both the experiments depicted in Fig. 6B and 6C show a time-dependent decrease of luciferase expression in untreated embryos, a result due to the progressive decrease of proliferative events during the time course of these studies.

Taken together these results demonstrate that anti-proliferative treatments decrease luciferase activity in MITO-Luc/GFP^1^ zebrafish, suggesting that this animal model represents a useful tool to screen the efficacy of candidate anti-proliferative drugs.

## Discussion

In this study we describe the generation and characterization of a cell proliferation reporter transgenic zebrafish line, called MITO-Luc/GFP, in which, using the activity of the master regulator of proliferation NF-Y, cell proliferation events in live fish can be detected. As a sensor of NF-Y transcriptional activity, we used a murine cyclin B2 promoter fragment, highly conserved between mouse and zebrafish, driving the expression of the GFP and luciferase genes.

The presence of two different reporter genes makes this line a versatile tool for cell proliferation imaging in embryo and adult animals.

Due to the optical transparency, cell proliferation can be analyzed in the whole-mount embryo by GFP^57^, while the detection of GFP in live adult animals is not achievable, due to the lack of skin transparency; therefore light detection is limited to organs and tissues near the body surface. In contrast, luciferase expression from inner organs and tissues can be detected in adult zebrafish^20^. Interestingly, the zebrafish transparent mutant strain^16^ if crossed with our strain, will make it possible to improve the signal-to-noise ratio and possibly enable the use GFP fluorescence also in adult zebrafish.

We generated two MITO-Luc/GFP zebrafish strains; MITO-Luc/GFP^1^ contains multiple insertions of the pT2KXIGΔin-cyclin B2-Luc/GFP construct for a highly detectable proliferation signal. The other strain, MITO-Luc/GFP^2^, is the result of a single insertion in the first intron of the AMP-activated, gamma 2 non-catalytic subunit protein kinase gene (prkag2a) on chromosome 24, position (28.173.305–28.302.316)(ZFIN Gbrowse). Both exhibit a similar behavior, but fluorescence and luminescence are lower in MITO-Luc/GFP^2^; thus, we focused our experiments on the MITO-Luc/GFP^1^ strain.

In this model, we observed that GFP and luciferase reporter genes are mostly expressed in tissues containing proliferating cells. This result was expected since the transcription of GFP and luciferase genes is under the control of a proliferation-dependent promoter that remains active during all phases of the cell cycle.

We here presented evidence that fluorescence and luminescence trace proliferation. We observed ubiquitous GFP expression in embryos from 6 hpf until 26 somite stage; in this period embryo cells divide continuously and GFP positive cells are proliferating cells. At 33 hpf GFP expression becomes more tissue specific and localizes in tissues known to contain a large number of actively proliferating cells^39^. It is important to acknowledge that GFP protein has a half-life of approximately 26 h^38^, therefore the fluorescent signal is the sum of all proliferative events that have characterized the embryo development. The long GFP protein half-life can partially mask the exact dynamic of proliferation events occurring during zebrafish embryogenesis. In contrast, the firefly luciferase protein has a half-life of about 3 h^39^ thus making this protein better suited to monitor the dynamicity of acute cellular events, such as proliferation waves. In agreement with GFP imaging, BLI analysis shows that the luciferase signal is widespread in embryos while it is localized in the abdomen of juvenile and adult fish. Moreover, the light emitted by 24 hpf embryos is higher than that emitted by juvenile and adult animals, indicating pervasive cell proliferation in embryos and more tissue- and organ-specific proliferation in juveniles and adults. Interestingly, in adult zebrafish luciferase activity of decreasing intensity was found in gonads, intestine, brain, kidney and heart; all organs known to contain proliferating cells^41–46^. Of note, the hematopoiesis is associated with kidney in these animals^14^ thus suggesting that ongoing hematopoiesis in this organ is the likely cause of the bioluminescence in the abdomen. Finally, by IHC and IF assays, we demonstrated that in specific tissues luciferase activity and GFP expression co-localize with proliferation markers pH3 and PCNA.

Taken together these results demonstrate that both GFP and luciferase reporter genes are expressed in proliferating cells in MITO-Luc/GFP reporter zebrafish, thus indicating that it may be a powerful tool to detect proliferation events in vivo.

To this end, we utilized different treatments to investigate changes in fluorescence or luciferase activity upon induction or inhibition of proliferation.

Caudal fin amputation in MITO-Luc/GFP^1^ 3 dpf embryos leads to a rapid migration of fluorescent cells, which proliferate in order to replace the wounded epidermis with new regenerating tissue. In adults light emission by the caudal fin is visible by BLI during the regeneration process. Unexpectedly, in parallel with the signal coming from caudal fin, we observed in the same fish a pronounced luciferase activity from the abdomen during longitudinal in vivo imaging sessions. Further, ex vivo experiments on dissected organs demonstrated high luciferase activity in the kidney. Interestingly, the kidney is a site of active hematopoiesis in adult zebrafish^14^ and it produces mature myeloid and lymphoid cells starting from 2 weeks of development^58^ thus indicating a strong correlation between luciferase activity and proliferation processes. It is noteworthy that the systemic biologic response to fin clip could only be highlighted in this model where it is possible to follow biological processes linked to proliferation in longitudinal experiments in the same animal. Taken together, these data clearly demonstrate that, after fin clip, GFP and luciferase expressions are induced in the MITO-Luc/GFP zebrafish line and strongly indicate that in this animal model it is possible to dynamically visualize induction of proliferation events in distinct body sites.

Finally, we obtained strong evidence of luciferase activity decrease upon anti-proliferative treatments. Sub lethal 5-FU, cell cycle inhibitors and X-ray, although to a different extent, inhibited luciferase expression in MITO-Luc/GFP embryos compared to untreated embryos.

In conclusion, the novel transgenic MITO-Luc/GFP zebrafish line is a read out for cell proliferation in vivo, and represents a powerful tool to follow the space and time evolution of proliferative events as a response to a variety of physiologic and pathologic conditions. Further, MITO-Luc/GFP zebrafish represents a highly promising, versatile, less expensive, easy-to-use and fast tool to screen anti- or pro-proliferative drug candidates in vivo. We expect that MITO-Luc/GFP zebrafish will complement experiments in cell lines^59^ and will be useful to readily and easily measure cell proliferation in several experimental applications, ranging from oncology to regenerative medicine. Moreover, MITO-Luc/GFP zebrafish, if crossed with zebrafish disease models, will allow in vivo imaging of fundamental physiologic and pathologic events including tumor progression and response to therapies.

## Materials and methods

### Zebrafish

Zebrafish (*Danio rerio*) embryos (6 hpf-72 hpf), juveniles (3–6 months) and adult (1 year—2 years) were raised and maintained under standard conditions in the fish facility of Cogentech s.c.a.r.l. (Aut. Prot. n. 007,894—29/05/2018), via Adamello 16—20,139 Milan (Italy) according to international (EU Directive 2010/63/EU) and national guidelines (Italian decree 4th March 2014, n. 26) on the protection of animals used for scientific purposes. All experimental procedures were approved by the FIRC Institute of Molecular Oncology Institutional Animal Care and Use Committee and Italian Ministry of Health. Adult density was maintained at maximum of 5 fish per liter in all experiments.

### Transgenic MITO-Luc/GFP zebrafish line

We used a described vector, pT2KXIGΔin^60^, for the generation of the MITO-Luc/GFP reporter constructs using standard cloning procedures. The constructs, pT2KXIGΔin-MITO-Luc/GFP, were obtained by cloning a murine cyclin B2 promoter fragment into the *BamHI* and *NcoI* sites of the pT2KXIGΔin plasmid. This murine cyclin B2 promoter fragment spans the – 266 to + 46 base-pair region respect to the transcription start site in front of a luciferase reporter and GFP reporter^22^. The construct was flanked by Tol 2 element sequences sites to allow transgenesis. Purified DNA was co-injected, at the concentration of 20 ng/μl, with 80 pg of Transposase mRNA into one-cell zebrafish embryos.

### Facs analysis on BrdU-treated embryos

About 200 MITO-Luc/GFP embryos were first incubated 3 h at 28.5° C, starting from 36 hpf, in E3 water containing 10 mM BrdU and 1% DMSO. Then, they were anesthetized, transferred in 2 ml tube and their cells dissociated with 0,25% trypsin solution. Cells were fixed in 2% formaldehyde. After incubation of 20 min on ice, fixed cells were centrifuged, washed with PBS + 1% BSA two times, resuspended in denaturing solution (2 N HCl) and incubated 25 min at RT. 0.1 M sodium borate was added for 2 min and two washes with PBS + 1% BSA were performed. Cells were incubated 1 h at room temperature with mouse anti-BrdU (1:5) and rabbit anti-GFP (1:500) in PBS + 1% BSA. Cells were then washed in 1 ml of PBS + 1% BSA, and resuspended in anti-mouse (conjugated wit Alexa-633) and anti-rabbit (conjugated wit Alexa-488) secondary antibodies and incubated 1 h at room temperature. Stained cells were washed once with 1 ml of PBS + 1% BSA and stored at 4° C. For FACS analysis, we used Attune NxT (Thermo Fisher Scientific, Waltham, MA, United States) instrument equipped with software FlwJo 10.7.1 (Vlaams Instituut voor Biotechnologie, Belgium and Babraham Institute in Cambridge, UK). As negative control, we analyzed cells incubated with primary antibodies only.

### DNA extraction and PCR analysis

Dechorionated embryos or fin fragments were lysate in Lysis Buffer (10 mM TrisHCl [pH 8.0], 1 mM EDTA, 0,3% Tween, 0,3% NP40), incubated for 10 min at 98 °C, cooled 3 min. in ice and leaved at 56 °C overnight with 1 mg/ml Proteinase K. Samples were extracted with phenol–chloroform adding NaAc 3 M pH 5.2, precipitated with 100% ethanol and collected by centrifugation. DNA pellets were washed with 70% ethanol and suspended in mQ water.

After genomic DNA extraction the positive transgenic fish were identified by PCR using the following primers:

Luciferase: oligonucleotide forward: 5′-ccggtactgttggtaaaatggaagacgcc -3′
  oligonucleotide reverse: 5′-cggacatttcgaagtattccgcgtacgtg -3′

PCR products were separated on a 2% agarose gel.

### Luciferase assay

Cells, embryos and organs were lysated in Lysis Reagent (Luciferase Assay Systems, *Promega*) and shaked for 10 min at room temperature. Lysates were then quick-freeze in dry ice, equilibrated to room temperature, and mixed with Luciferase Assay Reagent (Luciferase Assay Systems, *Promega*). The light produced during enzymatic reaction were measured by Victor lumiscence-reader (*Perkin Elmer*). Finally, the protein concentration for each sample was determined by Pierce BCA Protein Assay Kit (*Thermo Scientific*). The values were normalized, thanks to Albumin standard control, for protein amounts.

### Fluorescence imaging

GFP fluorescence emission was detected using the ST2 *Leica* stereo-microscope, imaged with a *Nikon* high resolution CCD camera and analyzed with the *Nikon* NIS-Elements software. Movies were achieved by merging sequential time laps acquisition, employed using a *Leica* TC-SP2 confocal microscope. To do this, embryos were anesthetized in a drop of 1% low-melting agarose containing 0.01 mg/l of Tricaine, placed into confocal dishes and subjected to sequential acquisitions with different duration and frequency, according to their stage of development.

### Bioluminescence imaging

For bioluminescence imaging, transgenic zebrafish (adult, juveniles and embryos) were incubated with 50 mM D-Luciferin, directly dissolved in the system water for 15 min, a timing sufficient to be ready for imaging. Adult zebrafish were then anesthetized in a low dose of tricaine (0.01 mg/ml) and placed on a water-soaked sponge support to reduce their stress^21^. Quantification of light emission was performed in photons/second and visualized in a pseudo color scaling. Time exposure was 5 min. Light emission was detected using the IVIS Lumina II CCD camera system and analyzed with the Living Image 2.20 software package (Caliper Life Sciences, License Serial number 0002–6485-2322–4101-4035). Photon emission was measured in specific regions of interest (ROIs). Data were expressed as photon/second/cm2/steradiant (p/s/cm2/sr)^22^. The intensity of bioluminescence was color-coded for imaging purposes; the scale used in each experiment is reported in each figure. For ex vivo BLI experiments, animals were sacrificed, dissected organs were immediately bathed in 50 mM D-Luciferin and subjected to a BLI session. Untreated zebrafish were used as negative control group. Six fishes were enrolled for each experimental group. Measurement of the enzymatic activity was done by bioluminescence, exposing the dissected organs to the CCD camera in a 96-well dish. Images of organs were detected as for the live animals.

### Immunofluorescence

Samples were fixed in 4% paraformaldehyde, rinsed 3 × in phosphate buffer saline (PBS), treated with 0,25% Tripsin, rinsed 3 times in Washing Buffer (1% Triton, 0.2% DMSO in PBS) and blocked for 1 h using Blocking Buffer (0,1% Triton, 1% DMSO, 5% normal goat serum in PBS). Samples were incubated overnight at 4 °C with polyclonal anti-phospho-H3 antibody (1:750, Abcam); polyclonal Mouse anti-firefly luciferase antibody (1:400, Abcam), monoclonal Rabbit anti-GFP antibody (1:100 Merk Millipore), or monoclonal Mouse anti-HuC/D (1:100, Santa Cruz). Samples were washed 5 × in Washing Buffer, blocked for 30 min using Blocking Buffer and incubated overnight at 4 °C with secondary antibody: anti-Rabbit Alexa488 (1:400); anti-Mouse Alexa546 (1:400); Alexa 633 (1:400) Samples were rinsed 2 times in Washing Buffer. Cells nuclei were stained with DAPI (Sigma-Aldrich).

### Immunohistochemistry

Adult fish were sacrificed by terminal anesthesia and fixed in 10 ml of 4% paraformaldehyde in PBS for 24 h. Samples were treated for two hours with I.E.D. Unit (Ion-Exchange Decal Unit, *BioCare Medical)* in order to decalcificate the tissues and then embedded in paraffin. Serial sections of 5 μm were mounted on slides, deparaffinized in xylene, and rehydrated. Sections were blocked for 1 h using Blocking Buffer (5% bovine serum albumin, 5% fetal bovine serum, PBS) and incubated overnight at 4 °C with polyclonal anti-phospho-H3 antibody (1:750, *Abcam*); polyclonal anti-firefly luciferase antibody (1:50, Novus Bio); monoclonal anti-GFP antibody (1:100, *Merk Millipore*) or monoclonal anti-PCNA antibody (1:100, *Abcam*). Sections were washed 3 times in Washing Buffer and processed following the manufacturer’s instruction of the MACH1 Universal HRP Kit (*Biocare*). The sections were stained with hematoxylin.

### Fin clip

For caudal fin clip injury, a fragment of about 50% of the caudal fin was removed from anesthetized fish using a sterile razor blade.

### Chemical treatments

24 hpf live dechorionated embryos were incubated for 6 h in a solution containing 100 mM 5FU in E3 water with 1% of DMSO at 28.5 °C then washed several times with fresh E3 water. Swimming embryos were then used to acquire in vivo a series of longitudinal images at different time points: before the start of chemical treatment, just after the end of the treatment and 6, 18, 24 and 42 h after the end of the treatment. Alternatively, for in vitro analysis, after the same treatment, anesthetized embryos were collected at the same time points and processed for the Luciferase Assay, as described before.

24 hpf live embryos were incubated for 6 h in a solution containing chemical treatments in E3 water with 1% of DMSO at 28.5 °C then washed several times with fresh E3 water. Then embryos were collected, lysated and subjected to Luciferase Assay, before the start of chemical treatment, just after the end of the treatment and 6, 18hrs later the treatment.

Compounds, features and concentration are listed.

- Nocodazole: microtubule destabilizer; 150 nM;
- Etoposide: topoisomerase II inhibitor; 20 mM.

### X-Ray treatments

Different amount of X-Rays (1.8 Gy and 2.7 Gy) were applied on 24 hpf transgenic embryos using X-Ray generator machine CP-160 (*Faxitron*), that can operate up to a power level of 160 kV. After 6 h, embryos were prepared and processed for the Luciferase Assay, as described before.

### Statistical analysis

Comparisons between two experimental groups were conducted using the unpaired two-tailed Student’s *t*-test. Comparisons between multiple experimental groups were conducted using ANOVA analysis. Differences between groups of data were taken statistically significant for *p* < 0.05. The *p*-values are represented as follows: (*) *p* > 0.05, (**) *p* < 0.01, (***) *p* < 0.001 and (****) *p* < 0.0001. All experiments were conducted at least in triplicate. All data are presented as the means ± SEM.

## Supplementary information

Supplementary Information.

Supplementary Video 1.

Supplementary Video 2.

Supplementary Video 3.

## Data Availability

The datasets generated and analysed during the current study are included in Supplementary Information files. Data regarding zebrafish model are available in the ZFIN repository, at the following link **Tg(ccnb2:Luciferase-GFP)**. Rest of the data are available from the corresponding authors on reasonable request.
