# Supplementary material for: MITO-Luc/GFP zebrafish model to assess spatial and temporal evolution of cell proliferation in vivo
Source: Sci Rep. 2021 Jan 12;11:671. doi: 10.1038/s41598-020-79530-5 (PMC7804000; doi:10.1038/s41598-020-79530-5)
Supplement: Supplementary file 1 — Supplementary Information. [file 41598_2020_79530_MOESM1_ESM.pdf]

MITO-Luc/GFP Zebrafish model to assess spatial and temporal evolution of cell proliferation *in vivo*

Luisa de Latouliere[1*], Isabella Manni[1*], Laura Ferrari[2], Federica Pisati[3], Maria Grazia Totaro[2], Aymone Gurtner[1,4], Emanuele Marra[5,6], Lucrezia Pacello[5], Ombretta Pozzoli[7,§], Luigi Aurisicchio[5,6], Maurizio C. Capogrossi[8,9], Gianluca Deflorian[2,10#], Giulia Piaggio[1#]

[1]UOSD SAFU, IRCCS - Regina Elena National Cancer Institute; Via Elio Chianesi 53, 00144, Rome, Italy.

[2]IFOM - FIRC Institute of Molecular Oncology, Milan, Italy

[3]Histopathology Unit, Cogentech S.c.a.r.l, Milan, 20139, Italy

[4]Institute of Translational Pharmacology, National Research Council, Rome, Italy

[5]Takis s.r.l. – Via Castel Romano 100, 00128, Rome, Italy

[6]VITARES -APS – Via Castel Romano 100, 00128, Rome, Italy

[7]Laboratorio di Biologia Vascolare e Medicina Rigenerativa - Centro Cardiologico Monzino - IRCCS (Istituto di Ricovero e Cura a Carattere Scientifico), Milan, Italy

[8]Johns Hopkins University School of Medicine, Division of Cardiology, Johns Hopkins Bayview Medical Center, 301 Building, Suite 2400, 4940 Eastern Avenue, Baltimore, MD 21224, USA

[9]Laboratory of Cardiovascular Sciences, National Institute on Aging / National Institutes of Health, Baltimore, MD 21224, USA

[10]Cogentech SRL - Benefit Corporation, Milan, Italy

* corresponding authors # last authors

§ present address: Pfizer Italia, Via A.M. Mozzoni 12, 20152, Milan, Italy

luisa.delatouliere@ifo.gov.it

isabella.manni@ifo.gov.it

Keywords: NF-Y, BLI, proliferation, imaging, zebrafish model

Running title: Imaging of proliferation in Zebrafish

SUPPLEMENTARY Table S1

Mouse and Zebrafish NFY subunits sequence alignments.

**Protein sequences alignment**	Zebrafish NF-YA NP_001076264.1			Zebrafish NF-YA like NP_001002731.1			Zebrafish NF-YBa NP_001017565.1			Zebrafish NF-YBb NP_001013340.1			Zebrafish NF-YC NP_955933.1		
Murine NF-YA NP_001104302.1	Query cover	E value	Perc. Ident.	Query cover	E value	Perc. Ident.	-			-			-		
	95%	3e-129	77,64%	76%	3e-135	82,18%									
Murine NF-YB NP_035044.1	-			-			Query cover	E Value	Perc. Ident.	Query cover	E value	Perc. Ident.	-		
							99%	6e-129	82,44%	99%	2e-128	80,58%			
Murine NF-YC NP_032718.2	-			-			-			-			Query cover	E value	Perc. Ident.
													97%	2e-177	81,07%

Summary table of mice and zebrafish protein sequence alignments of Nuclear Factor Y subunit A, B, and C. All zebrafish protein sequences present on Zebrafish May 2017 (GRCz11/danRer11) assembly available in UCSC Genome Browser (https://genome.ucsc.edu/, NCBI Ref Seq) are reported respect to those of Mouse Dec. 2011 (GRCm38/mm10) assembly. Query cover, E value and percent identity are reported for each alignment.

SUPPLEMENTARY FIGURES

Supplementary Figure S1

Murine cyclin B2 minimal promoter >mm10_ncbiRefSeqCurated_NM_007630.2 range=chr9:70421591-70421978

tatgtccttaggtgatggctccccactcctacccctaaaattaagacaatcttttccagtaggaggaaaaattatttt
atttaatatcagggactagaatttgaaaataagactgtagacaaggaaacaacaaagcctggtggcctcgctggttgc
Tatgacaagcaaatacaagccag**ccaat**caacgtgcagaaaggccttccagtctag**ccaat**gggttgcgcgcgccctg
cgtgcgtctac**ccaat**agtgcgtcagcggcgc**+1**GGTATTTGAATCGCGGACCGGGCGGTGGACGCGGAGCGGCGGGG
CCCTGACCCTCCCAACGGTGTCGCAGACCGGAGTGGCTGTGCCTCGTCCGCACTTGCCAGGGCGGCCCTC**ATG**

Zebrafish cyclin B2 like promoter >danRer11_ncbiRefSeqCurated_NM_199430.1 range=chr7:30626113-30626587

catacgcaagccttccaactttctctgatgatgagaacattgatttttttatatgtcctgtctttagatagcaatggt
ttttggaacagtgtcaacatcatattatactttatccctatttttttacagtctatgatccctagcctacttgcagtt
acaacttcaatcgttctcggatgaataaatgacgttacagacagagctacatgtgaaaccggagatgagggatgattg
ataagcgccgcctctcttttcat**ccaat**cgaactctagtatttcccgagccagtga**ccaat**cagagaacgcagagcgg
tatttgaatcaccagcaggcctttgtactgtatacgcacggcttgttattagttgagttggacgagaaacgcc**+1**AAA
CAAAGCTAAACGGCATTTTAAATTTTTTTATTTAATTTAACCGAGGCATCACCAAAAAGAATATTCATC**ATG**

Cyclin B2 minimal promoter sequences in mice and zebrafish. The two sequences are significantly different and present only minor similarities (https://blast.ncbi.nlm.nih.gov/), however, two of the three CCAAT boxes (underlined) present mice are conserved in zebrafish in terms of reciprocal position and distance from the ATG start codon. Promoter fragment (lower case), 5'UTR (capital case) and ATG start codon (bold) are shown (https://genome.ucsc.edu/, NCBI Ref Seq). The murine sequence from -266 to +46 bp was been used to generate MITO-Luc/GFP zebrafish (https://blast.ncbi.nlm.nih.gov/).

Supplementary Figure S2

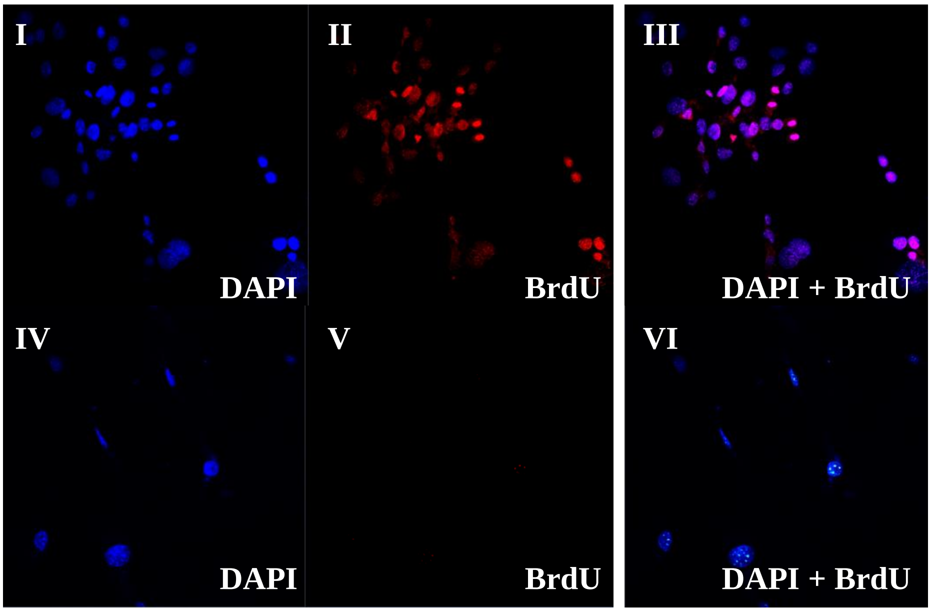

Immunofluorescence analysis of DAPI (I, IV), BrdU incorporation (II, V), and DAPI-BrdU merge (III, VI) in proliferative C2C12 (I,II,III) and in terminal differentiated C2C12 (IV,V,VI) after 3h BrdU pulse (20 μM final concentration). Proliferative C2C12 show 73% of BrdU positive cells while terminal differentiated C2C12 show 8% of BrdU positive cells rate respect to total cells number.

Supplementary Figure S3

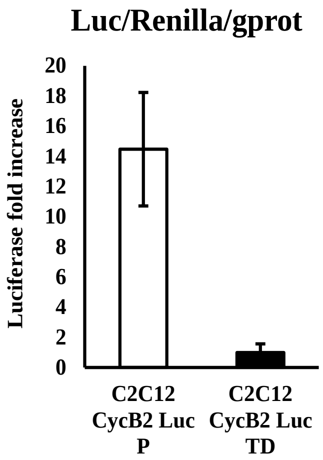

Relative luciferase activity obtained in pT2KXIGΔin-cylinB2-Luc/GFP transfected C2C12. Proliferative cells (P) show 14,5-fold increased transcriptional activity compared to terminal differentiated cells (TD). The error bars are mean standard deviations from two experiments performed in duplicate.

Supplementary Figure S4

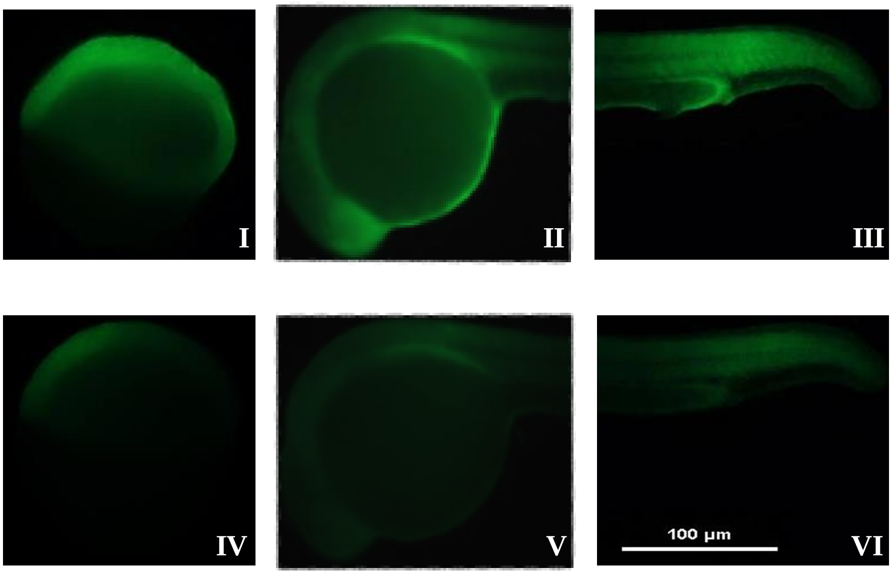

GFP expression in 6 hpf (I,IV) and 24 hpf embryos [head (II, V) and caudal fin (III,VI)] collected from MITO-Luc/GFP[1] (I,II,III) and MITO-Luc/GFP[2] (IV,V,VI) zebrafish lines. MITO-Luc/GFP[1] embryos exhibit higher fluorescence intensity than MITO-Luc/GFP[2]. The images were with a LeicaTC-SP2 confocal microscope.

Supplementary Figure S5

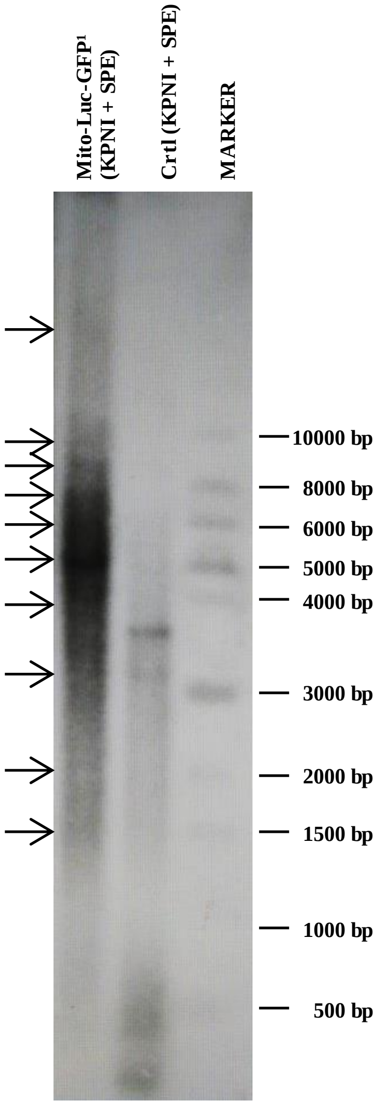

Southern Blotting analysis of the transgene insertion after *KpnI* and *SpeI* genomic digestion. Bands in the first line (black arrows) represent multiple transgene insertions in MITO-Luc/GFP[1] zebrafish; the band in the second line is a GFP transgenic zebrafish line used as control [Tg(kdrl: EGFP)]. The third lane shows molecular weight markers. Full-length blot is presented in Supplementary Figure S17.

Supplementary Figure S6

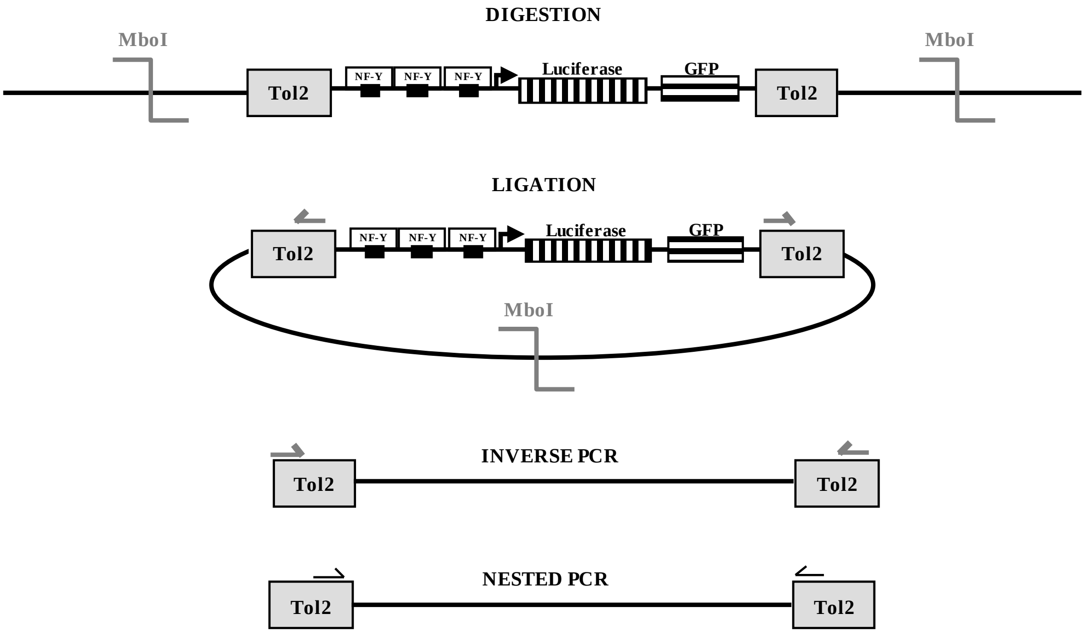

Schematic representation of Inverse PCR protocols.

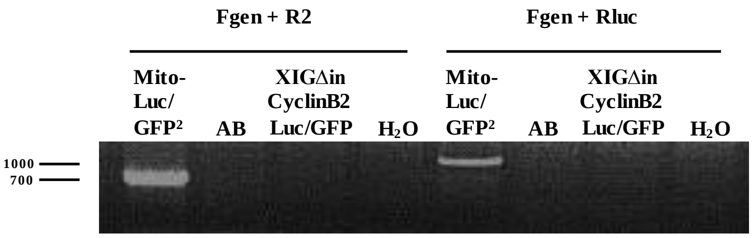

PCR with an oligonucleotide complementary to the genomic DNA flanking the transgene (forward) and two oligonucleotides complementary to the inserted transgene (reverse). Full-length gel is presented in Supplementary Figure S17.

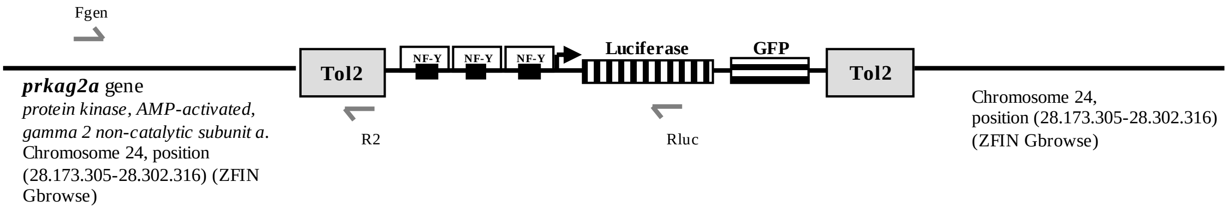

Schematic representation of the PCR.

Supplementary Figure S7

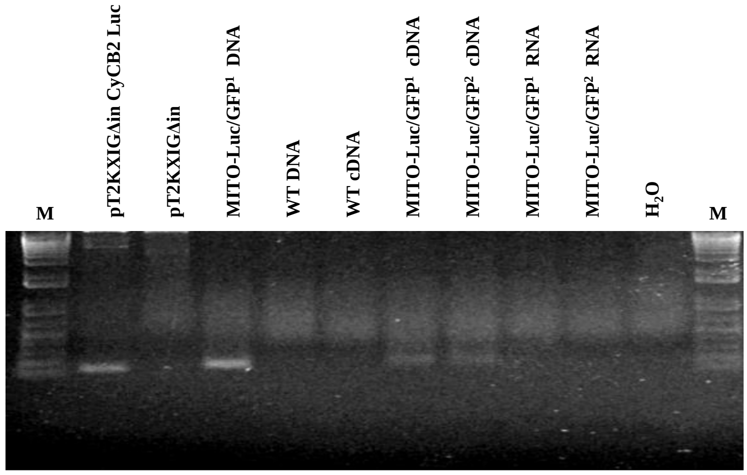

PCR on cDNA obtained from MITO-Luc/GFP zebrafish lines show that both transcripts are present on a single transcript because the oligonucleotide forward is designed on the Luciferase mRNA and the oligonucleotide reverse is designed on the GFP mRNA. The presence of the PCR amplified product indicates that the two mRNA are on a single transcript. From the left: molecular standard, pT2KXIGΔin-cylinB2-Luc/GFP plasmid as positive control, pT2KXIGΔin empty plasmid as negative control, MITO-Luc/GFP^{1} genomic DNA as positive control, AB wild type genomic DNA as negative control, AB wild type cDNA, MITO-Luc/GFP^{1} cDNA, MITO-Luc/GFP^{2} cDNA, MITO-Luc/GFP^{1} RNA, MITO-Luc/GFP^{2} RNA, PCR mix without DNA, molecular standard. Full-length gel is presented in Supplementary Figure S17.

Supplementary Figure S8

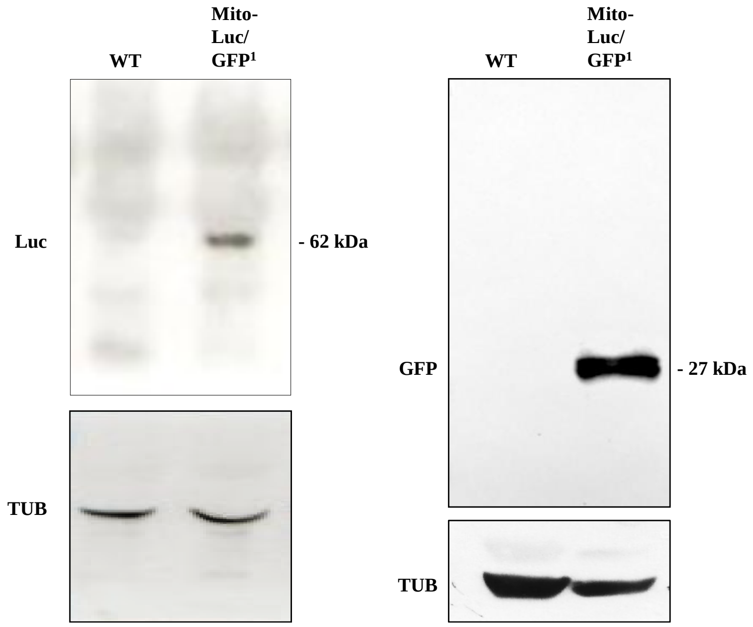

Western blotting analysis of wild type and MITO-Luc/GFP[1] zebrafish protein extracts. Full-length blots are presented in Supplementary Figure S17.

Supplementary Figure S9

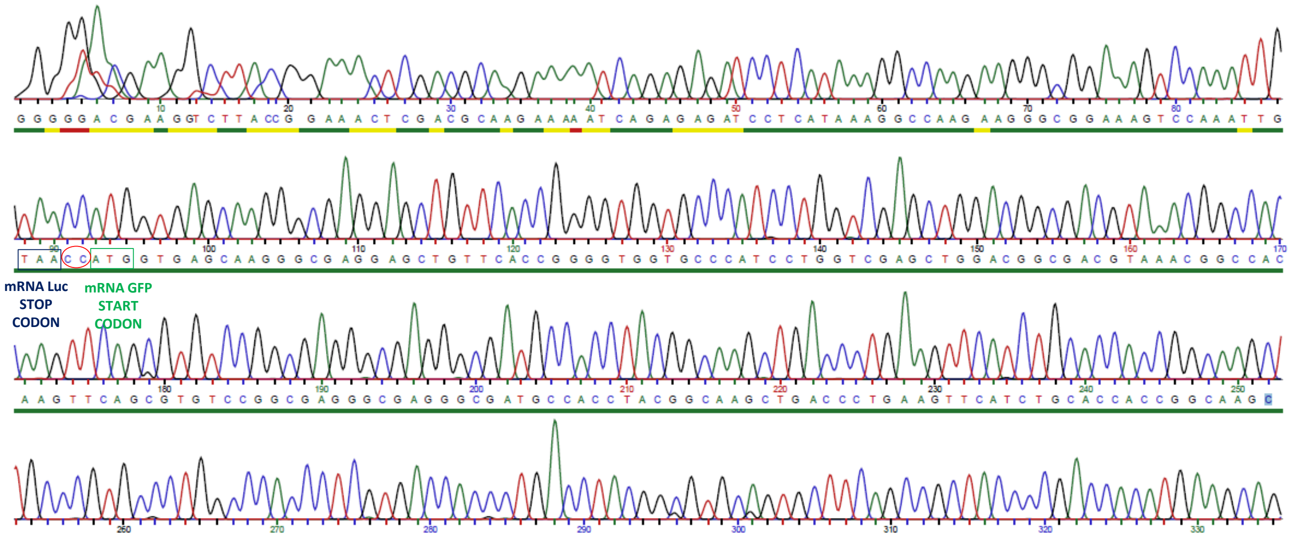

Sequencing of pT2KXIGΔin-cylinB2-Luc/GFP plasmid fragment between Luciferase and GFP transcripts. mRNAs are not in frame for the presence of two cytosine nucleotides (circled in red) between mRNA Luciferase stop codon (squared in blue) and GFP start codon (squared in green) as shown at the beginning of the second line.

Supplementary Figure S10

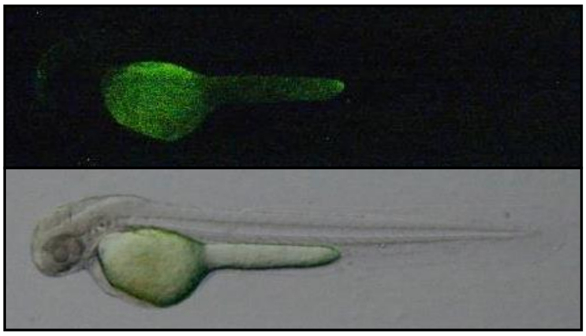

Autofluorescence analysis of AB 33 hpf embryo. Light coming from yolk is due to the auto-fluorescence. Bright light image is shown.

Supplementary Figure S11

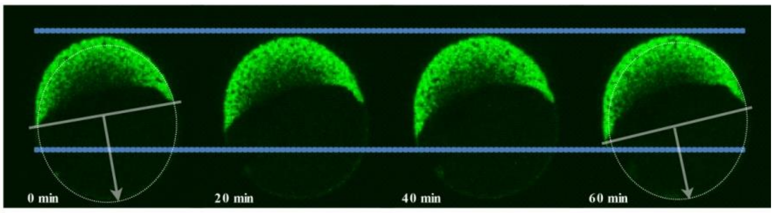

Representative frame pictures of time laps acquisition of 4.5 hpf MITO-Luc/GFP[1] embryo imaged every 4 min for 1,3 hrs using a Leica TC-SP2 confocal microscope. Embryo initially covers about 30% of yolk (embryo picture on the left, 0 min) but at the end of the imaging, thanks to the morphogenetic cell movement occurring during epiboly stages, it gets to cover about 50% of yolk (embryo picture on the right, 60 min). Dotted lines are shown as references, dotted circles highlight the shape of the yolk sack, white arrows indicate the direction of the morphogenetic movements and the distance between the growing embryo and vegetal pole.

Supplementary Movie S1

Time lap acquisition of 4 hpf MITO-Luc/GFP[1] embryo imaged every 4 min for 1,3 hrs using a LeicaTC-SP2 confocal microscope.

Supplementary Movie S2

Time lap acquisition of 19 hpf MITO-Luc/GFP[1] embryogenesis imaged every 25 min for 15 hrs using a a LeicaTC-SP2 confocal microscope.

Supplementary Figure S12

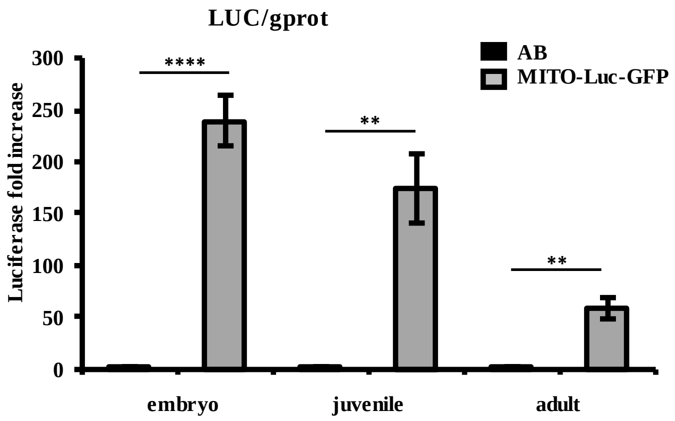

Luciferase activity in homogenate embryos, juveniles and adults from MITO-Luc/GFP[1] line and AB line as control. The error bars are mean standard deviations from three experiments. Asterisks denote significant differences between MITO-Luc/GFP[1] line and AB samples assessed by t-test (**p < 0.01, ****p < 0.0001).

Supplementary Figure S13

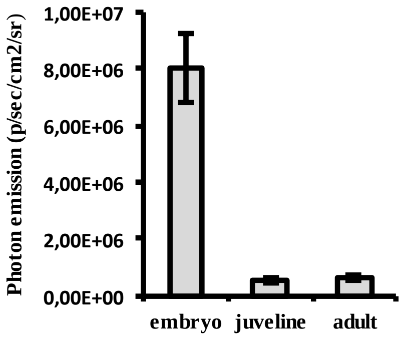

Photon emission quantification following BLI of individual MITO-Luc/GFP[1] zebrafish at 24 hpf embryo, juvenile and adult stages. The error bars are mean standard deviations from at least three independent experiments. All individual samples were imaged with the same color scale bar.

Supplementary Figure S14

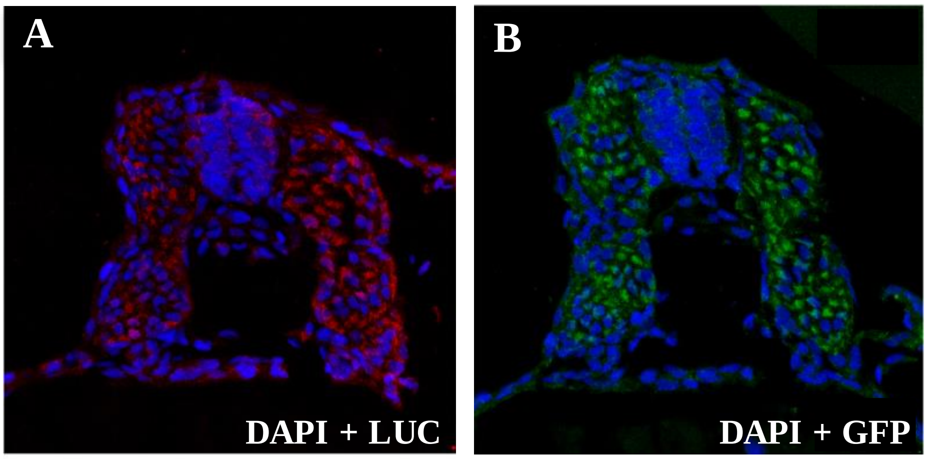

Immunofluorescence analysis of luciferase-DAPI **(A)** and GFP-DAPI merge **(B)** in 24 hpf embryo trunk. DAPI has been employed to visualize nuclei.

Supplementary Movie S3

Time lap acquisition of caudal fin regeneration in 3 dpf MITO-Luc/GFP[1] zebrafish embryos after fin clip procedure. Embryo was imaged every 15 min for 14 hrs using a LeicaTC-SP2 confocal microscope.

Supplementary Figure S15

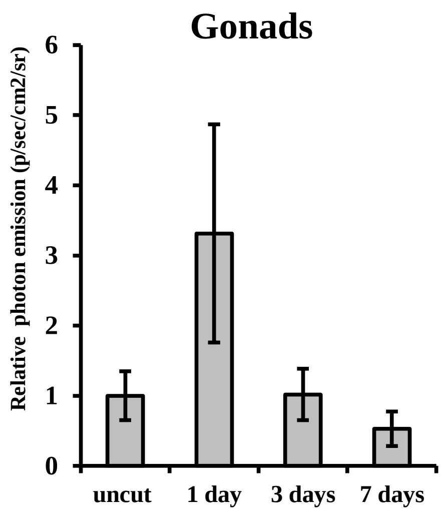

Relative quantification of *ex vivo* BLI of zebrafish gonads, both testes and ovaries, after fin clip. Luciferase activity was measured 1, 3, and 7 days after fin clip treatment. The error bars are mean standard deviations from six samples, three testes and three ovaries. The differences are not statistically significant.

Supplementary Figure S16

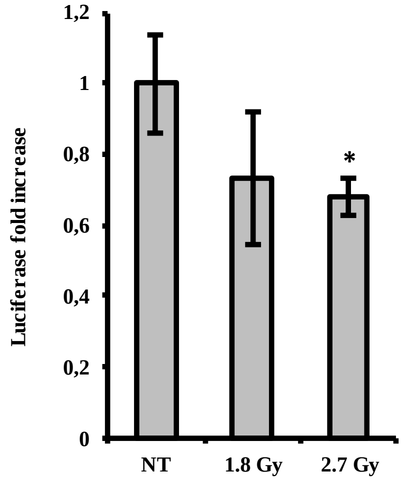

Luciferase activity of MITO-Luc/GFP[1] embryo pools 6 hrs after X-Ray treatment at the indicated doses. The error bars are mean standard deviations from three experiments performed in duplicate or in triplicate. Asterisks indicate significant differences between control and treated groups as assessed by t-test (*p < 0.05).

Supplementary Figure S17

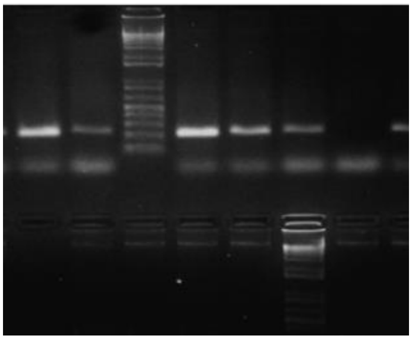

Full-length gel of Figure 1C.

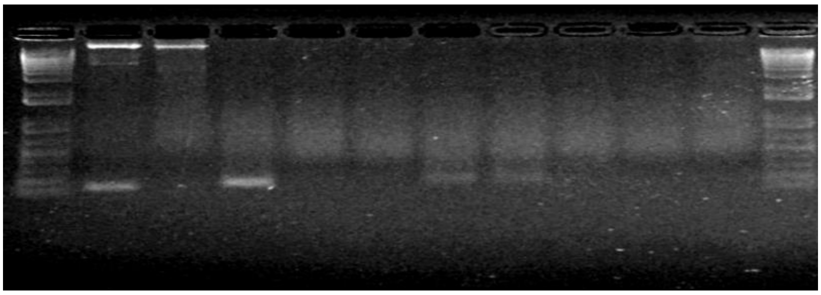

Full-length gel of Supplementary Figure S7.

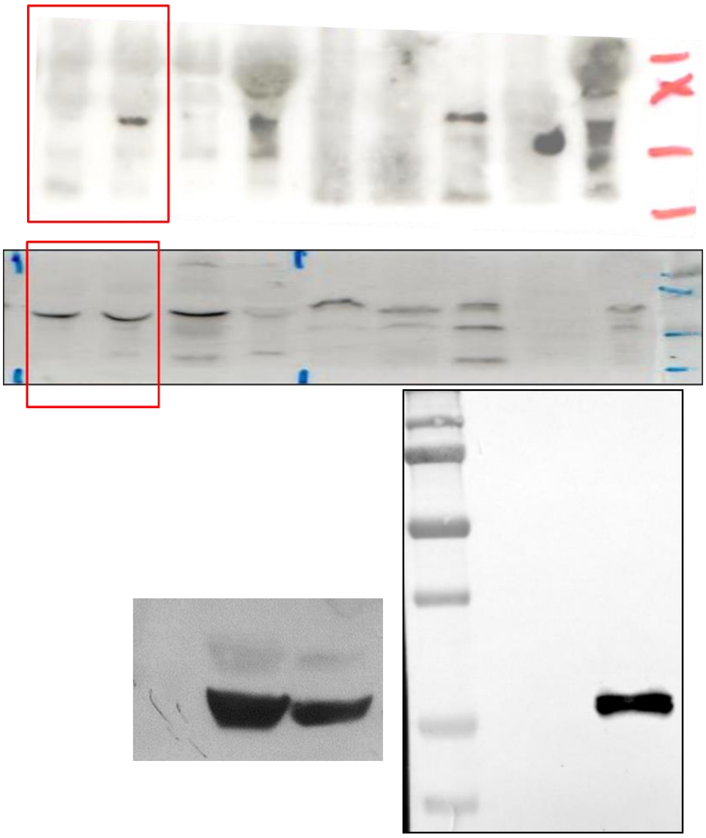

Full-length blots of Supplementary Figure S8.

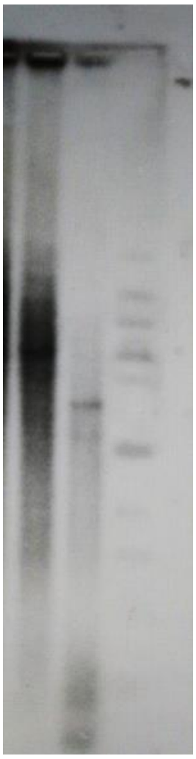

Full-length blot of Supplementary Figure S5.

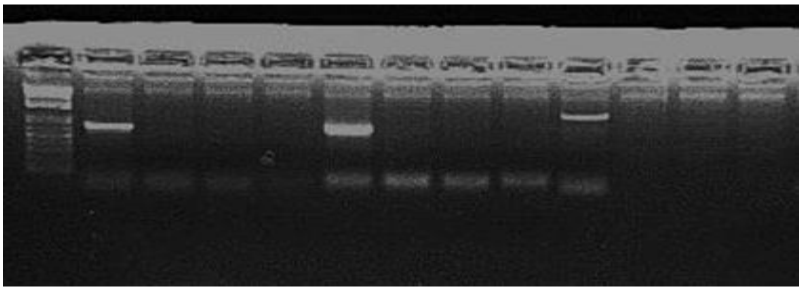

Full-length gel of Supplementary Figure S6.

SUPPLEMENTARY METHODS

C2C12 cell culture and cell tranfection

Immortalized mouse myoblast cell line, C2C12, were cultured in DMEM (Gibco BRL) supplemented with 10% FBS and antibiotics in a 5% CO2 humidified atmosphere. Cell were transfected with 14 mg of pT2KXIGΔin-cylin B2-Luc/GFP plasmid, using Lipofectamine LTX and PLUS reagent (Invitrogen) following the manufacturer's instructions. Transfection was done as cotransfections with a Renilla luciferase CMV-driven plasmid as internal control to check transfection efficiencies. 18h after, transfected cells were divided into two dishes. The first was grown in proliferating state for 48h under high serum conditions. In the second, differentiation was obtained by plating the cells into collagen-coated dishes under low serum conditions (DMEM supplemented with 5ug/ml insulin, 5 ug/ml transferrin) for 72 h. 50μM cytosine b-D-arabinofuranoside (Ara-C) was added to medium in the first 48h to eliminate undifferentiated cells and then removed [35]. At the end cells were collected and prepared for Luciferase assay. Reporter activity was measured using the dual luciferase assay system (Promega) according to the instructions of the manufacturers.

Immunofluorescence on BrdU-treated cells

BrdU administration was done directly in cell medium at 20 μM final concentration for 3 hours. Cells were fixed with 2% formaldehyde, permeated with 0,25% Triton X-100 and DNA was denatured with 2N HCl. Then cells were blocked 30min with 5% FBS, and subjected to staining using anti-BrdU (Roche), Cy3-conjugated donkey anti-mouse (Jackson Immuno Research Laboratories). Slides were analyzed within 24 h.

Fluorescence Imaging

Cell images were recorded by using a Zeiss LSM 510 Meta confocal laser scanning microscope equipped with a 40X and 60X/1.23 NA oil immersion objective. As laser (488 and 514 nm), and HeNe laser (543 nm) were used to excite the fluorophores. The LSM 510 R. 3.2 META (Zeiss) image analysis software was used.

RNA extraction and rt-PCR analysis

Embryos were collected in Trizol Reagent (Gibco) and homogenized by passing several times through a syringe. Once embryos were completely homogeneous RNAs were extracted following the manufacturer's instructions. RNAs were quantified and treated with DNAsi I (Invitrogen) to avoid DNA contamination. The first-strand cDNA was synthesized according to the instructions for the M-MLV RT kit (Invitrogen). PCR was performed using the following primers:

F Luc: oligonucleotide forward: 5′-GTTGCGCGGAGGAGTTGTG-3′

RGFP: oligonucleotide reverse: 5′-TCCTCGCCCTTGCTCACCAT-3′

The oligonucleotide forward is designed on the Luciferase mRNA, while the oligonucleotide reverse is designed on the GFP mRNA. The presence of the PCR amplified product indicates that the two mRNA are on a single transcript.

Western blot analysis

For protein extraction, embryos were homogenizated and boiled 10 minutes in a 250 mM TrisHCl lysis buffer (pH 6.8) containing 4% SDS and 10% glycerol. Proteins extracts were resolved by SDS-PAGE and electro transferred to nitrocellulose. Membrane was blocked with 5% non-fat dry milk for 1 h at room temperature and subsequently incubated with primary antibody for 16 h at 4°C. The following antibodies were used: anti GFP polyclonal (Merck Millopore), anti Luc polyclonal (Novus Bio) and anti-β actin (Sigma-Aldrich). Immunoreactivity was detected by sequential incubation with HRP-conjugated secondary antibody.

Inverse PCR

Genomic DNAs from both transgenic zebrafish lines were digested with *MboI* (BioLabs) restriction enzyme, having no recognition sites within the transgene insertion. Cleavage products were circularized using *T4 DNA ligase* (BioLabs) under condition that promote the formation of monomeric circle. After digestion, samples were hated to 65°C to inactivate restriction enzymes. Sample were precipitated with ethanol 100% adding 3M NaAc and amplificated with *Phusion High-Fidelit DNA Polymerase* (BioLabs) using primers synthesized within the transgene in the opposite orientations to those normally employed for PCR. Primers

for Inverse PCR are complementary to the opposite strand and orientated such that extension proceeds outward from the transgene. Reopen of the circularized molecules was not necessary. Nested primers were employed to further increase the specificity of PCR [60].

Following primers were synthesized:

Inverse PCR: oligonucleotide forward: 5′-gagaggctgcaaatagcagg -3’

oligonucleotide reverse: 5′-gagctaggcttgacactaac -3’

Nested PCR: oligonucleotide forward: 5′-ccctcggatatcacggg -3’

oligonucleotide reverse: 5′-cgttctactgaagttaaatctgagg -3’

Multiple PCR products were obtained from the MITO-Luc/GFP[1] line. Sequencing has not given reliable results, indicating the presence of multiple transgene insertions. Single PCR product was obtained from the MITO-Luc/GFP[2] line. Sequencing has permitted to determine the nucleotides sequence of genomic DNA flanking the transgene (24:28293209-28293338), allowing the insertion localization in the first intron (15909bp) of the *prkag2a* gene in the 24th chromosome of zebrafish genome.

To confirm achieved result, a forward primer was designed on the genomic DNA flanking the transgene founded by sequencing. The amplification of PCR product only in MITO-Luc/GFP[2] line by using reverse primer complementary to the insertion sequence validate the transgene localization.

Following primer was synthesized:

Fgenomic: oligonucleotide forward: 5′-ccctcggatatcacggg-3’

It was used with:

Inverse PCR: oligonucleotide reverse: 5′-gagctaggcttgacactaac-3’

Luciferase: oligonucleotide reverse: 5′-cggacatttcgaagtattccgcgtacgtg-3’

Southern Blotting

Genomic DNAs of MITO-Luc/GFP[1] line and a control GFP transgenic line [Tg(kdrl: EGFP)] were digested at 37°C over night with both *KpnI* and *SpeI* restriction enzymes (BioLabs). 10 µg of digested DNAs were run on a 1% agarose, 1X TAE gel at 50V for 4hrs without EtBr with Quick-Load 1 kb DNA Ladder, New England Biolabs as molecular weight marker. Gel was washed in Depurination solution (0.25M HCl) once for 15 min; in Denaturation solution (0.5M NaOH, 1.5M NaCl) once for 30 minutes and in Neutralizing solution (0.5M Tris pH 7.5, 1.5M NaCl) twice for 20 minutes each time. DNA was capillary transferred over night with 10x SSC (3M NaCl, 300mM Na-Citrate, pH 7) on nylon membrane (Amersham) by using a paper towels blot. Membrane was UV-crosslinked, pre-hybridized with PerfectHyb Plus Hybradization Buffer (Sigma) for 5 mins at 68°C and hybridized for 3 hrs with a radio-labeled GFP probe in the same buffer. Membrane was washed in Low Stringency wash (2X SSC, 0.1% SDS) once for 15 minutes, in High Stringency wash (0.5X SSC, 0.1% SDS) twice for 20 mins and in Ultra-High Stringency wash (0.1X SSC, 0.1% SDS) once for 20 mins. Membrane was exposed to a film with an intensifying screen.

SUPPLEMENTARY RAW DATAS

This file shows all raw data referring to the figures and supplementary figures presented in the paper.

The following tables are the raw data related to the figure indicated in each table legends.

The Student t-test has been used to determine if two sets of data were significantly different from each other.

cps	pT2KXIGΔin Luc/GFP	pT2KXIGΔin CyclinB2 Luc/GFP
	84	6382
	179	17276
	52	3494
	124	8940
means	109,75	9023
SEM	23,7128	2569,8203
fold	1	82,2141
p value		0,023896

Raw data related to figure 1B. Luciferase assay in one to four-cell stage AB embryos transiently injected with the pT2KXIGΔin-Luc/GFP or the pT2KXIGΔin-cylinB2-Luc/GFP. Data sets are significant different (*p<0,05).

cps	Mito-Luc/GFP[1]	Mito-Luc/GFP[2]
	1680	92
	1380	140
	1660	112
	1740	106
	1060	113
	1200	96
	1020	
means	1391,4286	109,8333
SEM	107,0462	6,3439
fold	12,66855	1
p value		6,17E-07

Raw data related to figure 1D. Luciferase activity in MITO-Luc/GFP[1] and MITO-Luc/GFP[2] zebrafish lines. Data sets are significant different (****p < 0.0001).

Photon emission (p/sec/cm²/sr)	background	heart	kidney	brain	intestine	ovary	testis
	4343000	4875000	5027000	5127000	6895000	18560000	222700000
	4290000	4575000	4659000	4991000	6452000	53980000	4112000
	2227000	4359000	4924000	5037000	7463000	16520000	105100000
	1404000	3511000	3366000	4006000	4616000		
	1419000	4033000	4465000	4084000	4746000		
	1473000	3987000	5075000	4352000	4734000		
	3550000						
	3202000						
	10920000						
	10570000						
	3252000						
	3358000						
	3108000						
	3396000						
	3392000						
means	3993600	4223333	4586000	4599500	5817667	29686667	110637333
SEM	722721	179988	238992	190188	472492	9929361	51571256

Raw data related to figure 3D. Photon emission quantification of organs extracted from adult zebrafish.

Photon emission (p/sec/cm²/sr)	fin uncut	fin 1 days	fin 7 days
	4989000	5151000	1947000
	4216000	12360000	4633833
	4074000	12280000	3208000
	3695000	11600000	3670000
	3619000	3526000	3946000
	4263000	3935000	3642000
means	4142667	8142000	3507806
SEM	183748	1622966	334592
fold	1	1,9654	0,8468
p value		0,049403	0,159919

Raw data related to figure 5D. Photon emission of fin from adult zebrafish after fin clip. t-test has been used to determine if data sets measured at 1 and 7 days after fin clip were significantly different from uncut fins data set. 1 days after fin clip data set is significant different from uncut data set (*p < 0.05).

Photon emission ($p/sec/cm^2/sr$)	intestine uncut	intestine 1 days	intestine 3 days	intestine 7 days
	6895000	6728000	8102000	2231000
	6452000	12230000	2244000	4352000
	7463000	12820000	4555000	3768000
	4616000	12390000	3484000	4155000
	4746000	4442000	4103000	5410000
	4734000	7962000	4481000	
means	5817667	9428667	4494833	3983200
SEM	472492	1316874	731381	461207
fold	1	1,6207	0,7726	0,6847
p value		0,040219	0,195619	0,034591

Raw data related to figure 5F_intestine. Photon emission of zebrafish intestines after fin clip. t-test has been used to determine if data sets measured at 1, 3 and 7 days after fin clip were significantly different from uncut zebrafish data set. 1 days and 7 days after fin clip data sets are significant different from uncut data set (*$p < 0.05$).

Photon emission ($p/sec/cm^2/sr$)	brain uncut	brain 1 days	brain 3 days	brain 7 days
	5127000	5044000	1607000	1569000
	4991000	13560000	1587000	3783000
	5037000	12390000	3563000	3673000
	4006000	12240000	3771000	4069000
	4084000	3658000	3859000	4175000
	4352000	4114000	3902000	
means	4599500	8501000	3048167	3453800
SEM	190188	1742836	421175	429301
fold	1,0000	1,8482	0,6627	0,7509
p value		0,069631	0,011953	0,044387

Raw data related to figure 5F_brain. Photon emission of zebrafish brains after fin clip. t-test has been used to determine if data sets measured at 1, 3 and 7 days after fin clip were significantly different from uncut zebrafish data set. 3 days and 7 days after fin clip data sets are significant different from uncut data set respectively with **$p < 0.01$ and *$p < 0.05$.

Photon emission (p/sec/cm^2/sr)	heart uncut	heart 1 days	heart 3 days	heart 7 days
	4875000	5276000	1734000	1519000
	4575000	12530000	2879000	3938000
	4359000	11920000	3316000	4278000
	3511000	11620000	3353000	4084000
	4033000	3709000	3428000	3492000
	3987000	3707000	3624000	
means	4223333	8127000	3055667	3462200
SEM	179988	1608637	257974	449687
fold	1	1,9243	0,7235	0,8198
p value		0,052306	0,006901	0,164876

Raw data related to figure 5F_heart. Photon emission of zebrafish hearts after fin clip. t-test has been used to determine if data sets measured at 1, 3 and 7 days after fin clip were significantly different from uncut zebrafish data set. 1 days and 3 days after fin clip data sets are significant different from uncut data set respectively with $*p < 0.05$ and $**p < 0.01$.

Photon emission (p/sec/cm^2/sr)	kidney uncut	Kidney 1 days	Kidney 3 days	kidney 7 days
	5027000	4748000	2167000	2329000
	4659000	11760000	1563000	3879000
	4924000	31420000	3527000	3496000
	3366000	11590000	10340000	3935000
	4465000	3669000	3553000	5303000
	5075000	5324000	3730000	4382000
	5240000	11941000	4633000	3194000
	3932000	10895000	3660000	
means	4586000	11418375	4146625	3788286
SEM	213294	2911310	886841	327163
fold	1	2,4898	0,9042	0,8261
p value		0,046001	0,659211	0,073649

Raw data related to figure 5F_kidney. Photon emission of zebrafish kidneys after fin clip. t-test has been used to determine if data sets measured at 1, 3 and 7 days after fin clip were significantly different from uncut zebrafish data set. 1 day after fin clip data set is significant different from uncut data set ($*p < 0.05$).

Cps fold	NT 0h	5FU 0h	NT 6h	5FU 6h	NT 18h	5FU 18h	NT 24h	5FU 24h	NT 42h	5FU 42h
	0,8026	0,4359	0,6305	0,2723	0,0891	0,0831	0,5609	0,2033	0,3923	0,4063
	0,9629	0,5078	1,1384	0,2220	0,1095	0,1046	0,4552	0,2384	0,4615	0,6109
	0,6259	0,3520	0,6455	0,5414	0,0993	0,0576	0,5121	0,2674	0,4719	0,5386
	0,7971	0,2040	0,7152	0,1437	0,2975	0,2447				
	0,9928	1,0356	0,4133	0,3447	0,8721	0,0775				
	1,5004	0,5391	0,5346	0,1181	0,8720	0,2894				
	1,1939	1,0454	0,3359	0,2639	0,3714	0,0818				
	1,0031	1,2765	0,9344	0,8197	0,5033	0,2416				
	0,8077	0,3749	1,0820	0,4098	0,2228	0,2001				
	0,7970	1,0043	0,8689	0,6393	0,3011	0,2592				
	0,6549	0,4637	1,0984	0,5082	0,1622	0,3640				
	0,8197	0,7801	0,8689	0,5246	0,2239	0,1559				
	1,2295	0,4853	0,4898	0,5082	0,5738	0,1887				
	0,8033	0,5228	0,6122		0,8525	0,1071				
	1,0492	0,3241	0,7347		0,3279	0,1258				
	0,6721	0,4109	0,7736		0,6230	0,2131				
	1,0492	0,2590	0,8585		0,2787	0,3443				
	0,8571	0,7049	0,7358		0,4590	0,1148				
	1,1020	0,6230	0,7075		0,8491	0,2295				
	0,8571	0,6885	0,6887		0,6415	0,0820				
	1,0000	0,4590	0,5943		0,5472	0,1803				
	1,1038	0,5902			0,6981					
	0,7830	0,4754			0,5566					
	0,9245				0,4811					
	0,7453				0,4340					
	0,8019				0,3585					
					0,3585					
means	0,9206	0,5897	0,7362	0,4089	0,4505	0,1783	0,5094	0,2363	0,4419	0,5186
SEM	0,0384	0,0562	0,0459	0,0547	0,0456	0,0193	0,0249	0,0151	0,0204	0,0489
p value		1,38E-05		0,000118		1,23E-05		0,001574		0,302938

Raw data related to figure 6B. Relative luciferase activity of MITO-Luc/GFP, untreated and 5FU treated, embryos that were collected at time 0, 6, 18, 24 and 42 hrs. To compare five independent experiments values are expressed as Cps fold relative to embryos before treatment onset. t-test has been used to determine if untreated and 5FU treated data sets were significantly different from each other. t-test was repeated for each experimental time. One-factor ANOVA analysis determines that F(44,085) > F crit(1,915) with a p-value of 1,92251E-47.

Cps fold	NT 0h	ETO 0h	NOC 0h	NT 6h	ETO 6h	NOC 6h	NT 18h	ETO 18h	NOC 18h
	0,8026	1,1696	0,7453	0,6305	0,6776	0,7358	0,0891	0,3368	0,3660
	0,9629	1,5564	1,0660	1,1384	0,6517	0,4811	0,1095	0,3673	0,3208
	0,6259	0,7677	0,7264	0,6455	0,4624	0,7170	0,0993	0,1858	0,1604
	0,7971	1,0866	0,9811	0,7152	0,4280	0,5000	0,2975	0,2092	0,3811
	0,9928	0,6211	0,6792	0,4133	0,3668	0,6132	0,8721	0,1320	0,2830
	1,5004	0,8878	0,9340	0,5346	0,3230	0,4340	0,8720	0,1342	0,2038
	1,1939	0,5738		0,3359	0,9344		0,3714	0,5082	0,4245
	1,0031	1,1639		0,9344	0,7049		0,5033	0,4590	0,1981
	0,8077	0,5082		1,0820	0,8852		0,2228	0,3115	0,2472
	0,7970	1,0072		0,8689	0,6393		0,3011	0,2951	
	0,6549	0,5082		1,0984	0,7213		0,1622	0,2295	
	0,8197	1,0164		0,8689	0,5246		0,2239	0,2951	
	1,2295	0,7347		0,4898	0,4898		0,5738		
	0,8033	0,6122		0,6122	0,3673		0,8525		
	1,0492	0,7347		0,7347	0,3673		0,3279		
	0,6721			0,7736			0,6230		
	1,0492			0,8585			0,2787		
	0,8571			0,7358			0,4590		
	1,1020			0,7075			0,8491		
	0,8571			0,6887			0,6415		
	1,0084			0,5943			0,5472		
	1,1038						0,6981		
	0,7830						0,5566		
	0,9245						0,4811		
	0,7453						0,4340		
	0,8019						0,3585		
							0,3585		
means	0,9206	0,8628	0,8553	0,7362	0,5696	0,5802	0,4505	0,2886	0,2872
SEM	0,0384	0,0747	0,0592	0,0459	0,0481	0,0476	0,0456	0,0328	0,0289
p value		0,462530	0,462402		0,022634	0,105857		0,034366	0,057344

Raw data related to figure 6C. Relative luciferase activity of MITO-Luc/GFP: untreated, Etoposide and Nocodazole treated, embryos were collected at time 0, 6 and 18 hrs. To compare five independent experiments values are expressed as Cps fold relative to embryos before treatment onset. t-test has been used to determine if any treated data set was significantly different from untreated data set. t-test was repeated for each experimental time. One-factor ANOVA analysis determines that F(19,647) > F crit(2,011) with a p-value of 4,51595E-19.

Luciferase/Renilla	C2C12 CycB2 Luc P	C2C12 CycB2 Luc TD
	0,3054293	0,0278474
	0,1413348	0,0030211
means	0,2233821	0,0154342
SEM	0,058016158	0,008777438
fold	14,473161	1

Raw data related to supplementary figure S3. Luciferase activity obtained in pT2KXIGΔin-cylinB2-Luc/GFP transfected C2C12. Proliferative cells (P) show 14,5-fold increased transcriptional activity compared to terminal differentiated cells (TD).

LUC/g prot	embryo AB	embryo MITO	juvenail AB	juvenail MITO	adult AB	adult MITO
	0,0277	4,3581	0,0252	6,3589	0,0193	0,9781
	0,0228	6,0864	0,0238	1,9164	0,0232	0,7113
	0,0198	7,3027	0,0199	4,1812	0,0182	1,3634
		7,3298		3,1315		2,1628
		3,5336		5,4280		0,6292
		5,1047		7,9332		1,3370
				4,6120		
				0,5857		
				1,9034		
means	0,0234	5,6192	0,0229	4,0056	0,0202	1,1970
SEM	0,0019	0,5818	0,0013	0,7423	0,0012	0,2099
fold	1,0000	239,7338	1,0000	174,5826	1,0000	59,1714
p value		0,000543		0,017920		0,010050

Raw data related to supplementary figure S12. Luciferase activity in homogenate embryos, juveniles and adults from MITO-Luc/GFP[1] line and AB line as control. t-test has been used to determine if data sets measured in MITO-Luc/GFP[1] and AB samples were significantly different from each other. t-test was repeated for each development stage. All MITO-Luc/GFP[1] data sets are significant different from AB wild-type data sets.

Photon emission (p/sec/cm^2/sr)	embryo	juvenails	adults
	1282000	478000	613000
	3480000	521000	526000
	3938000	598000	747000
	4946000		
	6519000		
	7375000		
	8234000		
	8522000		
	8630000		
	9006000		
	9339000		
	15530000		
	17590000		
means	8030077	532333	628666
SEM	1206881	28660	52481

Raw data related to supplementary figure S13. Photon emission quantification following BLI of individual MITO-Luc/GFP[1] zebrafish at 24 hpf embryo, juvenile and adult stages.

Photon emission (p/sec/cm^2/sr)	gonad uncut	gonad 1 days	gonad 3 days	gonad 7 days
	222700000	75740000	102000000	13380000
	4112000	32300000	10320000	50970000
	105100000	110800000	21970000	15580000
	50430000	11380000	49610000	13890000
	15010000	696000000	247200000	140100000
	133180000	831600000	109000000	
means	88422000	292970000	90016667	46784000
SEM	30845900	137454236	32414492	21814610
fold	1	3,3133	1,0180	0,5291
p value		0,21449091	0,97468690	0,36200887

Raw data related to supplementary figure S15. Photon emission of zebrafish gonads after fin clip. t-test has been used to determine if data sets measured at 1, 3 and 7 days after fin clip were significantly different from uncut zebrafish data set. No data set is statistically significant different from uncut data set.

cps	NT	1.8 Gy	2.7 Gy
	1040	1320	720
	1760	680	1080
	1600	320	960
	880	1560	840
	840	1360	840
	1720	640	1120
	1280	360	880
means	1303	891	920
SEM	138	179	50
fold	1	0,6842	0,7061
p value		0,117597	0,032612

Raw data related to supplementary figure S16. Luciferase activity of MITO-Luc/GFP[1] embryo pools 6 hrs after X-Ray treatment at the indicated doses. t-test has been used to determine if any Xray dose data set was significantly different from untreated data set. 2,7 Gy dose data set is significant different from untreated data set (*$p < 0.05$). Though One-factor ANOVA analysis does not show significant different between groups means [determines that F(2,536) < F crit(3,554) with a p-value of 0,1071].
